# Astrocytic ET‐1 System Determines Microglia Phenotype Following Spinal Cord Injury

**DOI:** 10.1002/advs.202507215

**Published:** 2025-05-30

**Authors:** Bingqiang He, Si Xu, Mengdi Li, Hui Li, Shaolan Li, Li Niu, Honghua Song, Rixin Cai, Yue Zhou, Zhilong Cao, Yingjie Wang, Yongjun Wang

**Affiliations:** ^1^ Key Laboratory of Neuroregeneration of Jiangsu and Ministry of Education Co‐innovation Center of Neuroregeneration Nantong University 19 Qixiu Road Nantong 226001 P.R. China; ^2^ Medical School of Nantong University 19 Qixiu Road Nantong 226001 P.R. China; ^3^ Department of Burn and Plastic Surgery Affiliated Hospital of Nantong University 20 Xisi Road Nantong 226001 P.R. China

**Keywords:** thrombin, endothelin‐1, astrocytes, microglia, phenotype

## Abstract

Microglia/macrophages accumulate at the lesion site by switching toward pro‐inflammatory (M1)‐dominant phenotype at the acute phase following spinal cord injury (SCI). Such biased polarization shapes the functional outcomes by expanding tissue damage. In the present study, the astrocytic endothelin‐1 (ET‐1) system is revealed to be immediately activated after SCI, driving microglia polarization toward M1, but suppressing toward M2 phenotype through activation of transcription coactivator YAP via ET_A_ and ET_B_ receptors. In addition, the activation of astrocytic ET‐1 system results in elevation of blood plasma ET‐1 level, suggesting a high diagnostic value. SCI‐induced thrombin is pinpointed as a crucial activator of the astrocytic ET‐1 system. The serine protease dramatically promotes the astrocytic expression of preproendothelin‐1 (ppET‐1) through protease‐activated receptor‐1 (PAR‐1)/RhoA/NF‐κB and PAR‐1/MAPKs/NF‐κB signal pathways. Meanwhile, it induces the expression of astrocytic endothelin‐converting enzyme 1 (ECE‐1) responsible for mature ET‐1 processing. Pharmacological inhibitors of PAR‐1 and ET‐1 are shown to be highly efficient in microglia M1 phenotype reversion and favorable for the recovery of rat locomotor function after SCI. The findings have revealed a novel mechanism of M1 microglia/macrophages swarming at lesion sites at the acute phase following SCI, and provide potential therapeutic approaches for neuroinflammation by targeting the astrocytic ET‐1 system.

## Graphical abstract

The study reveals that astrocytic ET‐1 system is solely activated by thrombin following SCI via RhoA/NF‐κB and MAPKs/NF‐κB signal pathway. The release of astrocytic ET‐1 drives microglia polarization toward M1 phenotype through YAP signaling via ET_A_ and ET_B_ receptors. Both PAR‐1 and ET‐1 inhibitors are highly efficient in reversing microglia M1 polarization, promoting recovery of rat locomotor function after SCI.

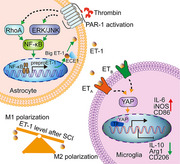

## 1 Introduction

Spinal cord injury (SCI) activates resident microglia and recruits peripheral monocytes at lesion site, where they mediate progressive neuropathology known as the secondary tissue damage.^[^ ^1^ ^]^ Synchronous to the pathological process, these cells experience sequential alterations in phenotypes, along with functional and molecular changes.^[^ ^1^, ^2^ ^]^ Heterogeneous microglia/macrophages are classified into classically‐activated M1 pathological phenotype, alternatively‐activated anti‐inflammatory M2 phenotype, and several transition states with overlapping molecular signatures at different phases following SCI.^[^ ^3^ ^]^ A dynamic shift in M1 and M2 phenotype at lesion site shapes the functional outcomes of the damaged spinal cord.^[^ ^1^, ^2^, ^4^ ^]^ During the acute phase after SCI, M1 phenotype microglia/macrophages dominate the landscape for one week, whereas M2 phenotype is only transiently present, due to a rapid transition toward M1.^[^ ^2^, ^4^ ^]^ Many phenotype inducers, especially the microenvironmental cues, are responsible for the conversion of M1 or M2 microglia/macrophages. For example, proinflammatory cytokines TNF‐α, IFN‐γ, IL‐6, and lipocalin‐2 are able to synergistically or differentially promote polarization of M1 phenotype.^[^ ^5^ ^]^ Conversely, activation of microglia/macrophages with IL‐4 or IL‐13 drives cell polarization toward M2 phenotype.^[^ ^1^, ^2^, ^5^ ^]^ To date, the key regulatory molecules that control phenotype switching toward M1 at acute phase following SCI remain unclear.

Endothelin system is one of the important feedback regulators under pathological condition, which consists of three endothelin isoforms (ET‐1, ET‐2, and ET‐3), two G‐protein‐coupled receptors (ET_A_ and ET_B_), and two activating peptidases (ECE1 and ECE2).^[^ ^6^ ^]^ Being originally identified in the endothelial cells of the vascular system, endothelins have been shown to mediate a variety of vascular pathophysiologies.^[^ ^7^ ^]^ Distinctively, the astrocytic ET‐1 system is predominantly activated in traumatic and neurodegenerative central nervous system (CNS) disorders, which aggravates the primary pathology by causing secondary damage.^[^ ^8^ ^]^ Although the ET‐1 can be synthesized by vascular endothelial cells in the CNS, it is only released toward the basolateral side, acting on smooth muscle cells to affect vasoconstriction.^[^ ^7^, ^9^ ^]^ As such, the astrocytes‐derived ET‐1 is recognized as an important player in mediating neurodeleterious effects, such as neuronal death, astrogliosis, impairment of fast axonal transport, and increase of blood‐brain barrier (BBB) permeability by targeting CNS parenchymal cells.^[^ ^8^, ^10^ ^]^ Indeed, an appropriate input of exogenous ET‐1 to the astrocytic system or to the normal CNS can result in severe neurological deficits,^[^ ^11^ ^]^ recapitulating the key roles of astrocytic ET‐1 system in driving neuropathological progression of CNS. Previous investigations point out that ET‐1‐mediated CNS damages are closely correlated with the inflammatory response.^[^ ^12^ ^]^ It is already known that the microglia/macrophages inducibly express both ET‐1‐specific ET_A_ and ET_B_ receptors,^[^ ^13^ ^]^ implying that the astrocytic ET‐1 system may contribute to the activation of inflammatory cells, including mediating the polarization of microglia M1 phenotype following CNS insults.

Mechanistically, the active endothelin peptide ET‐1 is produced through a set of complex molecular processes. The preproendothelin gene is regulated by several nuclear transcription factors, such as c‐fos, nuclear factor‐1, AP‐1, and GATA‐2, followed by the synthesis of the preproendothelin.^[^ ^14^ ^]^ Then, it is cleaved at dibasic sites by furin‐like endopeptidases to form big endothelin.^[^ ^15^ ^]^ This biologically inactive intermediate is further cleaved by endothelin‐converting enzymes (most prominent ECE1/2) to produce mature ET‐1.^[^ ^16^ ^]^ Once being released from activated cells, the ET‐1 can exert its biological effects via ET_A_ and/or ET_B_ receptors for prolonged time periods even its disassociation from the receptors.^[^ ^7^, ^17^ ^]^ Such unique property of ET‐1 renders it unfavorable for ongoing tissue/organ recovery if not resolved immediately. In the vascular endothelin system, some soluble factors including TNF, interleukins (ILs), insulin, norepinephrine, angiotensin II, and glucose have been revealed to stimulate ET‐1 production from endothelial cells.^[^ ^18^ ^]^ However, there are hardly any clues available in the activation of astrocytic ET‐1 system following CNS damage. Under physiological conditions, the astrocytes do not produce ET‐1. So, the molecules involved in regulating astrocytic ET‐1 might be more specific for particular types of pathology.^[^ ^8a^ ^]^ Recently, the serine protease thrombin has been highlighted as a potential activator of astrocytic ET‐1 system, as it strongly promotes gliosis, production of inflammatory mediators, and formation of glial scar.^[^ ^19^ ^]^ Thrombin‐mediated cell events of astrocytes are achieved through enzymatic cleavage of PAR‐1, PAR‐3, and PAR‐4 receptors to induce intracellular formation of inositol‐1,4,5‐trisphosphate (IP3), with a concomitant activation PKC and mobilization of intracellular Ca^2+^ that are essential for ET‐1 production.^[^ ^8^, ^20^ ^]^ To explore the activating mechanism of astrocytic ET‐1 system, as well as the potential effects on microglial M1 phenotype switch following SCI, the rat spinal cord contusion was established and the active state of astrocytic ET‐1 system was examined at lesion site. SCI‐induced elevation of thrombin protein was shown to significantly stimulate the production of astrocytic ET‐1, which in turn determined the M1‐dominant phenotype of microglia at acute phase through activating transcription coactivator YAP. Our results have unveiled a novel mechanism of astrocytic ET‐1 system‐mediated neuropathology, which provides the alternative therapeutic targets for neuroinflammation following SCI.

## 2 Results

### 2.1 Astrocytic ET‐1 is Predominantly Induced at Lesion Site and Correlates with Microglial M1 Polarization Following SCI

**Figure 1:**
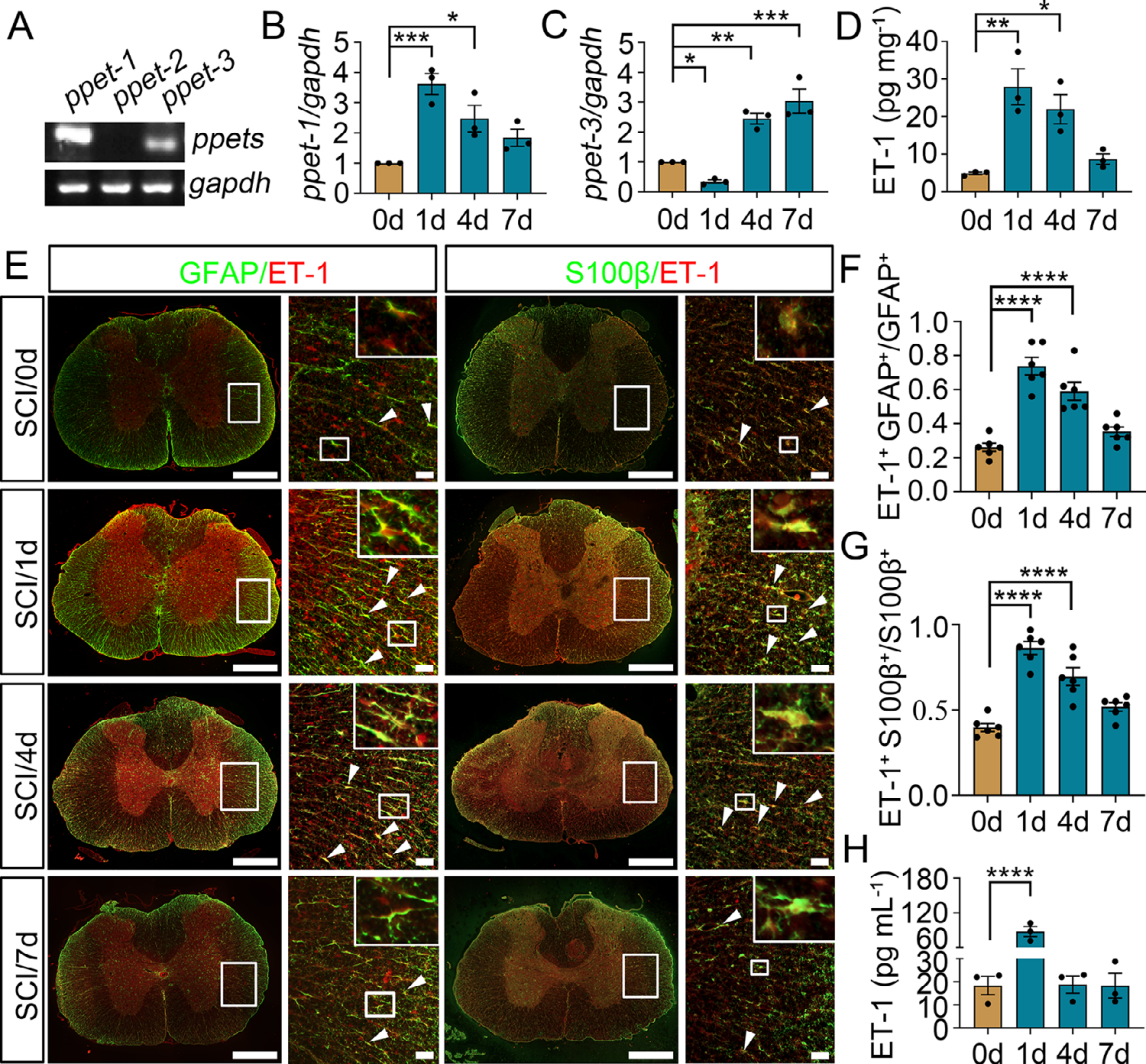
Examination of astrocytic ET‐1 system activation following rat SCI. A) Semi‐quantitative PCR assay determined the transcriptional levels of ppET‐1, ppET‐2 and ppET‐3 in the intact spinal cord. B, C) qRT‐PCR assays for transcriptional levels of ppET‐1 and ppET‐3 at 0 d, 1 d, 4 d and 7 d following SCI. Data are expressed as mean ± SEM, *n* = 3, ^*^ *p* < 0.05, ^**^ *p* < 0.01, ^***^ *p* < 0.001, one­way analysis of variance followed by Dunnett's *post hoc* test. Quantities were normalized to endogenous *gapdh*. D) ELISA measurement of mature ET‐1 protein at lesion sites at 0 d, 1 d, 4 d and 7 d following SCI. Data are expressed as mean ± SEM, *n* = 3, ^*^ *p* < 0.05, ^**^ *p* < 0.01, one­way analysis of variance followed by Dunnett's *post hoc* test. E) Immunostaining showed colocalization of ET‐1 with GFAP‐ and S100β‐positive astrocytes at 0 d, 1 d, 4 d and 7 d following SCI. Rectangle indicates region magnified. Arrowheads indicate the positive signals. Scale bars, 500 µm and 50 µm in magnification. F, G) Quantification data as shown in (E). Data are expressed as mean ± SEM, *n* = 6, ^****^ *p* < 0.0001, one­way analysis of variance followed by Dunnett's *post hoc* test. H) ELISA measurement of plasma levels of ET‐1 in peripheral blood at 0 d, 1 d, 4 d and 7 d following SCI. Data are expressed as mean ± SEM, *n* = 3, ^****^ *p* < 0.0001, one­way analysis of variance followed by Dunnett's *post hoc* test.

To gain a better understanding of SCI‐activated subtypes of the astrocytic endothelin system, the constitutive expression of three endothelin isoforms in the spinal cord was first analyzed. Semi‐quantitative PCR results displayed that the preproendothelin‐1 (ppET‐1) was predominantly, while ppET‐3 slightly, and ppET‐2 deficiently expressed in the cord tissue under physiological condition (**Figure** **1**A). In addition, the ppET‐1 expression was significantly upregulated at 0 d, 1 d, 4 d, and 7 d following SCI, while the ppET‐3 was remarkably elevated at 4 d and 7 d (Figure 1B,C). ELISA for mature ET‐1 peptide in the damaged tissues showed a consistent expression profile with that of ppET‐1 expression (Figure 1D). Thus, ET‐1 may represent a critical contributor of the endothelins in the modulation of neuropathology after SCI. Next, we sought to examine the specific cell types for mature ET‐1 production. Immunostaining demonstrated that the GFAP‐ and S100β‐positive astrocytes were the primary cells responsible for the inducible production of ET‐1 (Figure 1E‐G). However, other cells including neurons, microglia, or endothelial cells displayed undetectable changes in ET‐1 abundance at 0 d, 1 d, 4 d, and 7 d following SCI (Figure S1, Supporting Information). To assess whether the activation of astrocytic ET‐1 system can be reflected by blood plasma parameters, ET‐1 levels in the blood plasma of the subjects were accordingly determined by ELISA. It was shown that the activation of astrocytic ET‐1 system could result in dramatic elevation of ET‐1 levels in the blood plasma at 1 d after SCI (Figure 1H). The data indicate that the astrocytic ET‐1 system is predominantly activated in response to SCI and can be indicated by the blood plasma parameters.

**Figure 2:**
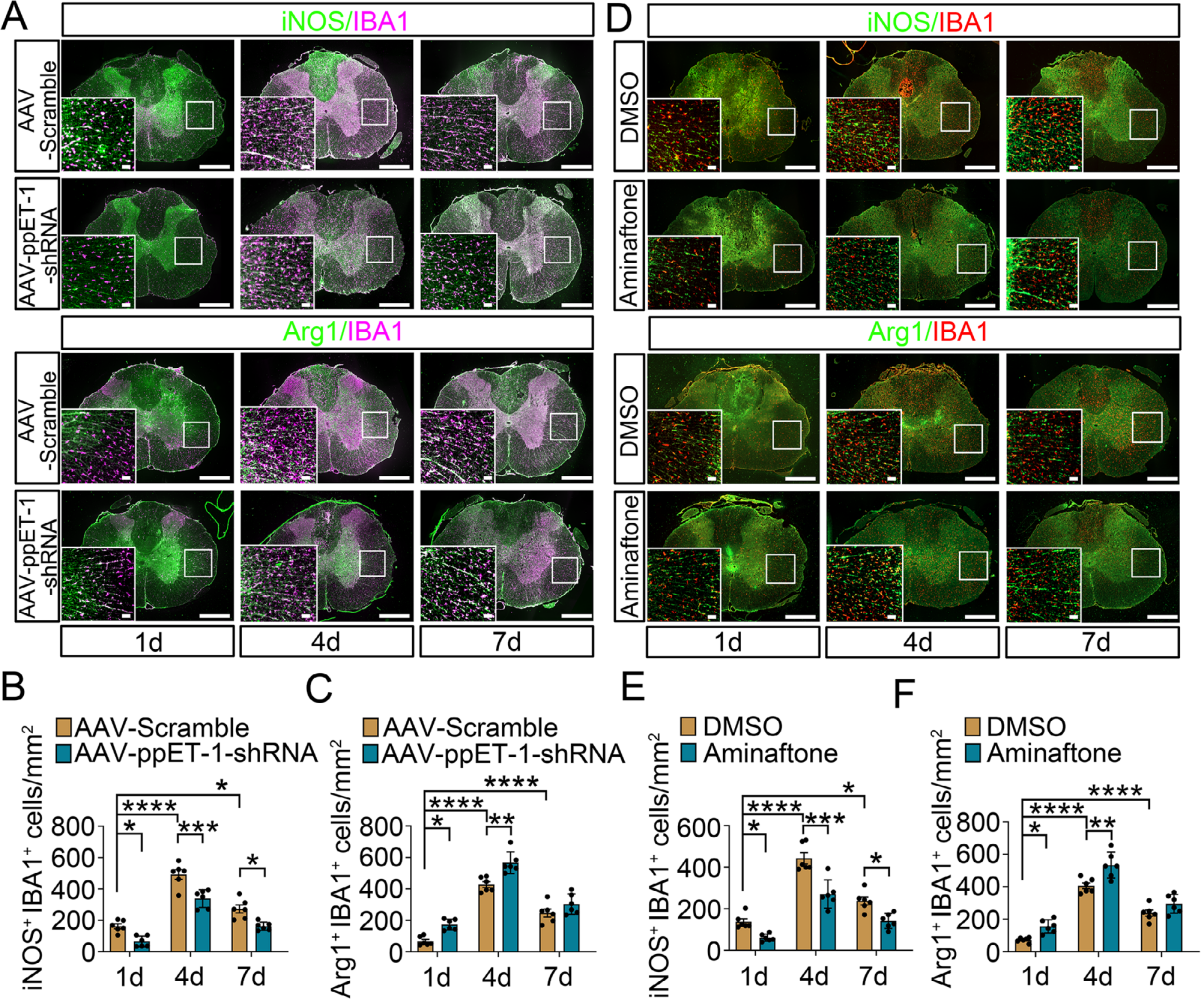
Correlation analysis between astrocytic ET‐1 system and polarization of microglia phenotypes following SCI. A) Immunostaining of iNOS^+^ IBA1^+^ and Arg1^+^ IBA1^+^ cells at the lesion site of the cord at 1 d, 4 d and 7 d, respectively, following SCI, after pre‐injection of 10 µL 1 × 10^13^ vg mL^−1^ AAV5‐GFAP‐mCherry‐ppET‐1‐shRNA or AAV5‐GFAP‐mCherry‐Scramble for 21 d. The rectangle indicates the region magnified. Scale bars, 500 µm and 50 µm in magnification. B, C) Quantification data as shown in (A). Data are expressed as mean ± SEM, *n* = 6, ^*^ *p* < 0.05, ^**^ *p* < 0.01, ^***^ *p* < 0.001, ^****^ *p* < 0.0001, two‐way analysis of variance followed by Sidak's *post hoc* test. D) Immunostaining of iNOS^+^ IBA1^+^ and Arg1^+^ IBA1^+^ cells at the lesion site of the cord at 1 d, 4 d and 7 d, respectively, following injection of 4.5 µL ET‐1 inhibitor Aminaftone (500 µg kg^−1^). The DMSO (0.1%) was used as vehicle. The rectangle indicates the region magnified. Scale bars, 500 µm and 50 µm in magnification. E, F) Quantification data as shown in (D). Data are expressed as mean ± SEM, *n* = 6, ^*^ *p* < 0.05, ^**^ *p* < 0.01, ^***^ *p* < 0.001, ^****^ *p* < 0.0001, two‐way analysis of variance followed by Sidak's *post hoc* test.

To determine the relations between astrocytic ET‐1 system activation and polarization of microglia phenotypes, the M1/M2 phenotypes were detected using the specific markers iNOS and CD86 for M1, Arginase 1 and CD206 for M2 at 1 d, 4 d, and 7 d following SCI. As the ET‐1 performs a wide range of CNS‐controlled functions as a neuropeptide,^[^ ^8^, ^21^ ^]^ both AAV5‐mediated knockdown of astrocytic ppET‐1 and pharmacological inhibitor of ET‐1, rather than extreme gene deletion, were used to examine the potential effects of ET‐1 on microglial phenotype alterations. Injection of 10 µL AAV5‐GFAP‐mCherry‐ppET‐1‐shRNA (1 × 10^13^ vg mL^−1^) or scramble at surgical site before 21 d of SCI, resulted in astrocytes‐specific expression (>91%) of exogenous mCherry (Figure S2A,B, Supporting Information). Accordingly, the expression of ET‐1 in the astrocytes at 1 d, 4 d, and 7 d following SCI, was markedly reduced by the virus transfection (Figure S2C–E, Supporting Information). Immunostaining revealed that the proportion of iNOS^+^ or CD86^+^ microglia significantly increased at 1 d, 4 d, and 7 d following SCI, in parallel with those of ET‐1 expression (**Figure** **2**A,B; Figure S3A,B, Supporting Information). Meanwhile, the number of Arginase1^+^ or CD206^+^ microglia also increased at lesion site from 4 d onward (Figure 2A,C; Figure S3A,C, Supporting Information). However, the astrocytes‐specific knockdown of ppET‐1 by AAV5 transfection significantly reduced the proportion of iNOS^+^ or CD86^+^ microglia, but increased those of Arginase1^+^ or CD206^+^ microglia at 1 d and 4 d (Figure 2A–C; Figure S3A–C, Supporting Information). An intrathecal injection of 4.5 µL ET‐1 inhibitor Aminaftone (500 µg kg^−1^) at lesion site displayed consistent results with those of AAV5‐GFAP‐mCherry‐ppET‐1‐shRNA (Figure 2D–F; Figure S3D–F, Supporting Information). The data indicate that the SCI‐activated astrocytic ET‐1 system is involved in microglial M1 polarization.

### 2.2 ET‐1 Drives Microglia Polarization Toward M1 Phenotype via ET_A_ and ET_B_ Receptors

**Figure 3:**
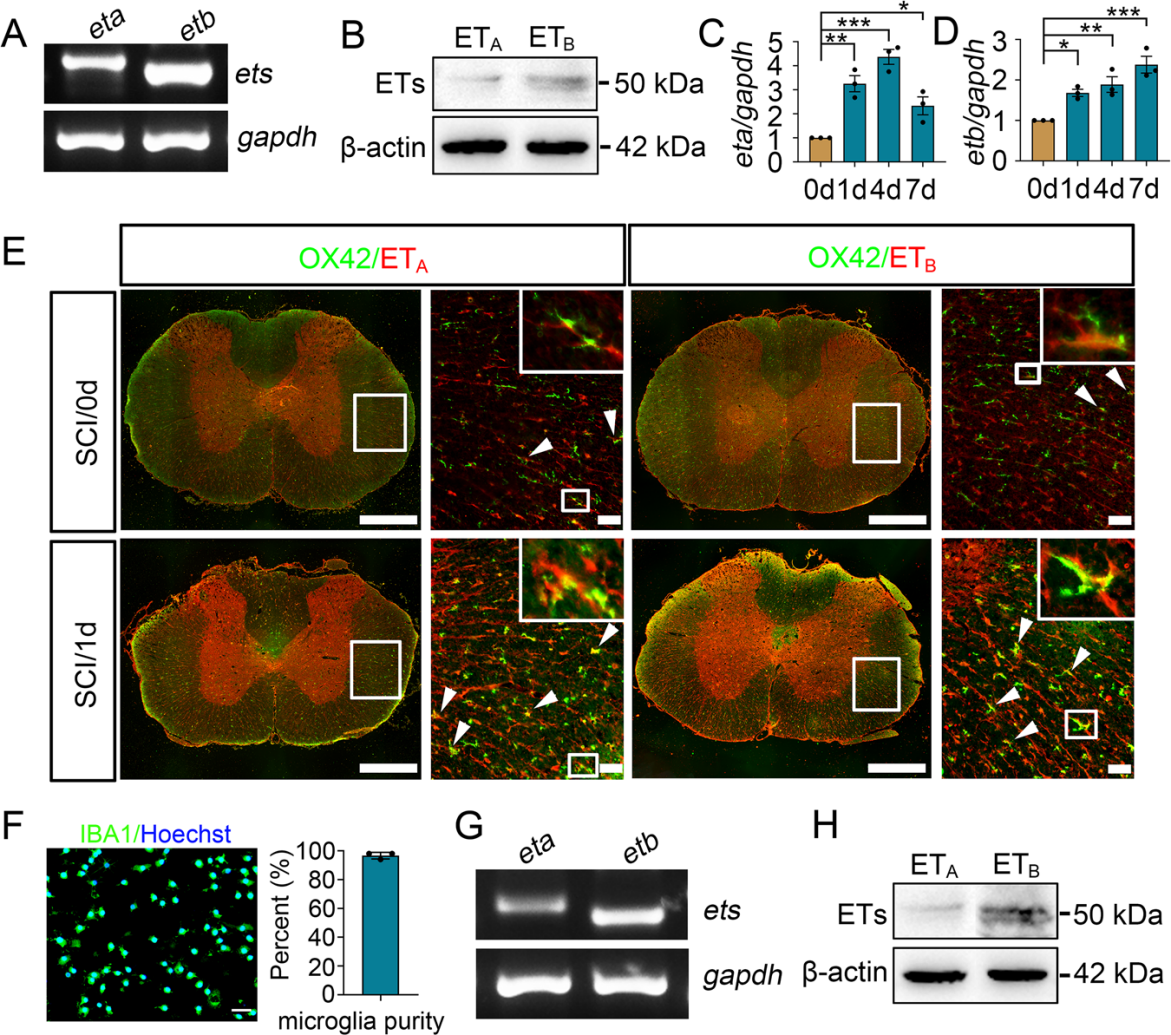
Detection of ET_A_ or ET_B_ receptors in microglia following SCI. A) Semi‐quantitative PCR examined the expression of ET_A_ and ET_B_ in the intact spinal cord. Quantities were normalized to endogenous *gapdh*. B) Western blot analysis of the protein levels of ET_A_ and ET_B_ in the intact spinal cord. Quantities were normalized to endogenous β‐actin. C, D) qRT‐PCR assays for transcriptional levels of ET_A_ (C) and ET_B_ (D) at 0 d, 1 d, 4 d and 7 d following SCI. Quantities were normalized to endogenous *gapdh*. Data are expressed as mean ± SEM, *n* = 3, ^*^ *p* < 0.05, ^**^ *p* < 0.01, ^***^ *p* < 0.001, one­way analysis of variance followed by Dunnett's *post hoc* test. E) Immunostaining showed colocalization of ET_A_ and ET_B_ receptors with OX42‐positive microglia before (0 d) and after SCI (1 d). Rectangle indicates the region magnified. Arrowheads indicate the positive signals. Scale bars, 500 µm and 50 µm in magnification. F) Isolation and purification of primary microglia from rat spinal cord, with purity over 95%. G) Semi‐quantitative PCR analyzed the expression of ET_A_ and ET_B_ in the primary microglia. Quantities were normalized to endogenous *gapdh*. H) Western blot analysis of ET_A_ and ET_B_ protein levels in the primary microglia. Quantities were normalized to endogenous β‐actin.

As ET‐1 mediates a variety of cell events through binding with two major receptor subtypes ET_A_ and/or ET_B_ in a tissue‐specific manner,^[^ ^7^, ^22^ ^]^ the dynamic expression of ET_A_ and ET_B_ in microglia was therefore detected before or after SCI. Determination of constitutive expression of the two subtypes revealed that the ET_B_ had a higher abundance than ET_A_ receptor in the spinal cord under physiological condition (**Figure** **3**A,B), and all of them displayed a significant upregulation at 1 d, 4 d and 7 d following SCI (Figure 3C,D). Immunostaining demonstrated that both ET_A_ and ET_B_ subtypes were distributed in the OX42‐positive microglia, with an elevated expression after injury (Figure 3E). Subsequently, the primary microglia were cultured and validated the existence of both receptors in the cells (Figure 3F‐H).

**Figure 4:**
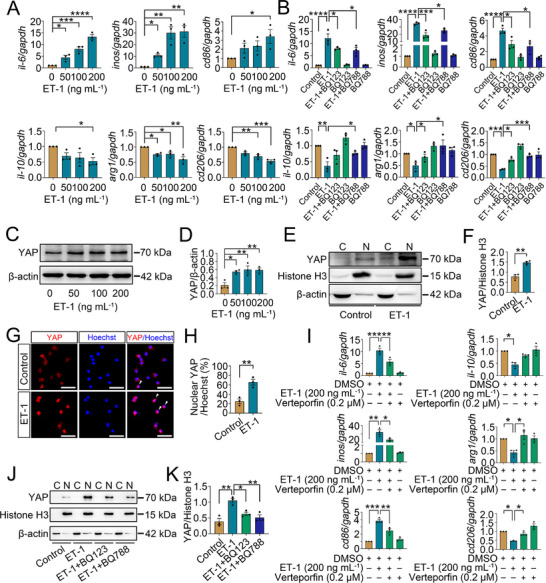
Effects of ET‐1‐induced YAP on the microglia M1 polarization. A) qRT‐PCR determination of transcriptional levels of M1 phenotype‐related genes IL‐6, iNOS and CD86, and M2 phenotype‐related genes IL‐10, Arg1 and CD206 following microglia challenged with 0–200 ng mL^−1^ rET‐1 for 24 h. Data are expressed as mean ± SEM, *n* = 3, ^*^ *p* < 0.05, ^**^ *p* < 0.01, ^***^ *p* < 0.001, ^****^ *p* < 0.0001, one­way analysis of variance followed by Dunnett's *post hoc* test. Quantities were normalized to endogenous *gapdh*. B) qRT‐PCR assays for M1 and M2 phenotype‐related molecules following microglia treated with 1 µM ET_A_ inhibitor BQ123 or 1 µM ET_B_ inhibitor BQ788 for 24 h in the presence of 200 ng mL^−1^ rET‐1. Data are expressed as mean ± SEM, *n* = 3, ^*^ *p* < 0.05, ^**^ *p* < 0.01, ^***^ *p* < 0.001, ^****^ *p* < 0.0001, one­way analysis of variance followed by Sidak's *post hoc* test. Quantities were normalized to endogenous *gapdh*. C) Western blot analysis of YAP after stimulation of the cells with 0–200 ng mL^−1^ rET‐1 for 24 h. D) Quantification data as shown in (C). Data are expressed as mean ± SEM, *n* = 3, ^*^ *p* < 0.05, ^**^ *p* < 0.01, one­way analysis of variance followed by Dunnett's *post hoc* test. Quantities were normalized to endogenous β‐actin. E) Western blot analysis of YAP in the cytoplasm and nucleus following microglia treatment with 200 ng mL^−1^ rET‐1 for 24 h. F) Quantification data as shown in (E). Quantities were normalized to endogenous β‐actin (cytoplasm) or Histone H3 (nucleus). Data are expressed as mean ± SEM, *n* = 3, ^**^ *p* < 0.01, two‐tailed unpaired Student's *t*‐test. G) Immunostaining showed the distribution of YAP in the cytoplasm and nucleus of the microglia following treatment with 200 ng mL^−1^ rET‐1 for 24 h. Arrowheads indicate YAP‐positive nucleus. Scale bars, 50 µm. H) Quantification of YAP‐positive nucleus co‐stained with Hoechst 33 342 as shown in (G). Data are expressed as mean ± SEM, *n* = 3, ^**^ *p* < 0.01, two‐tailed unpaired Student's *t*‐test. I) qRT‐PCR analysis of M1 and M2 phenotype‐related genes following the cells treated with 0.2 µM YAP inhibitor Verteporfin for 24 h in the presence of 200 ng mL^−1^ rET‐1. Data are expressed as mean ± SEM, *n* = 3, ^*^ *p* < 0.05, ^**^ *p* < 0.01, ^***^ *p* < 0.001, one­way analysis of variance followed by Sidak's *post hoc* test. Quantities were normalized to endogenous *gapdh*. J) Western blot analysis of YAP in the cytoplasm and nucleus following microglia treatment with 1 µM ET_A_ inhibitor BQ123 or 1 µM ET_B_ inhibitor BQ788 for 24 h in the presence of 200 ng mL^−1^ rET‐1. K) Quantification data as shown in (J). Quantities were normalized to endogenous β‐actin (cytoplasm) or Histone H3 (nucleus). Data are expressed as mean ± SEM, *n* = 3, ^*^ *p* < 0.05, ^**^ *p* < 0.01, one­way analysis of variance followed by Sidak's *post hoc* test.

To ascertain the promoting effect of ET‐1 on microglia polarization toward M1 phenotype, the primary microglia were stimulated with 0−200 ng mL^−1^ recombinant ET‐1 protein (rET‐1) for 24 h. Determination of M1 and M2 phenotype‐related characteristic molecules at transcriptional levels revealed that the rET‐1 was efficient in upregulating the M1 (IL‐6, iNOS, and CD86) while downregulating the M2 phenotype‐related genes (IL‐10, Arg1, and CD206) in a dose‐dependent manner (**Figure** **4**A). Additionally, exposure of the microglia to 200 ng mL^−1^ rET‐1 in the presence of 0.5 µg mL^−1^ LPS or 20 ng mL^−1^ IL‐4 for 24 h, gave rise to a synergistic effect of rET‐1 in LPS‐mediated expression of M1, but an inhibitory effect in IL‐4‐mediated expression of M2 phenotype‐related genes (Figure S4, Supporting Information).

Next, the specific inhibitor of ET_A_ (BQ123) and ET_B_ (BQ788) was applied to analyze the precise receptor subtype of ET‐1 action on microglia. MTT assay excluded the toxicity of the inhibitors to the cells (Figure S5A,B, Supporting Information). Results revealed that treatment of the microglia with 1 µM ET_A_ inhibitor BQ123 or 1 µM ET_B_ inhibitor BQ788 for 24 h in the presence of 200 ng mL^−1^ rET‐1 was able to significantly attenuate the effects of rET‐1 on promoting M1 and inhibiting M2 phenotype‐related molecules of the cells (Figure 4B). The data indicate that ET‐1 drives microglia polarization toward M1 phenotype via ET_A_ and ET_B_ receptors.

### 2.3 ET‐1 Switches Microglia Toward M1 Phenotype Through Activation of YAP Signaling

ET‐1 signaling is very complicated due to activating classical and non‐classical signaling pathways, which are converged to transcriptional factors including c‐FOS and c‐MYC.^[^ ^7a^ ^]^ To elucidate the regulatory mechanism of ET‐1 in promoting microglia polarization toward M1 phenotype, the cells were stimulated with 0–200 ng mL^−1^ rET‐1 for 24 h, followed by Western blot analysis of critical transcription factors c‐FOS and c‐MYC. The results demonstrated that the rET‐1 was able to induce the expression of c‐MYC, rather than that of c‐FOS (Figure S5C–E, Supporting Information). To gain an insight into the possible role of c‐MYC in the ET‐1‐mediated M1 polarization of microglia, the cells were treated with 10 µM c‐MYC inhibitor 10058‐F4 in the presence of 200 ng mL^−1^ rET‐1 stimulation. Unexpectedly, the 10058‐F4 was inefficient in reversing the expression of M1 phenotype‐related genes, though it had a slight effect on IL‐6 expression (Figure S5F,G, Supporting Information), implying a novel regulatory mechanism of ET‐1 in mediating microglial M1 phenotype conversion. The transcription coactivator in the Hippo pathway,^[^ ^23^ ^]^ Yes‐associated protein (YAP), was then taken into consideration, as targeting YAP has been found to alleviate acute liver injury and inflammatory bowel disease by changing macrophage polarization.^[^ ^24^ ^]^ Western blot demonstrated that exposure of the microglia to 0–200 ng mL^−1^ rET‐1 for 24 h significantly increased the protein levels of the YAP (Figure 4C,D). Analysis of YAP subcellular localization revealed that the rET‐1 also promoted the YAP translocation from cytoplasm to the nucleus, as was detected by immunoblot of cytosolic and nuclear fraction and immunostaining (Figure 4E‐H).

To explore the role of YAP signaling in ET‐1‐mediated microglia polarization, the YAP inhibitor Verteporfin was applied to treat the cells in the presence of rET‐1 stimulation. The inhibitor was nontoxic to the microglia (Figure S5H, Supporting Information). Treatment of the cells with 0.2 µM Verteporfin was shown to remarkably attenuate the inducible effects of 200 ng mL^−1^ rET‐1 on the expression of M1 phenotype‐related genes IL‐6, iNOS and CD86, while the inhibitory effects on the M2 phenotype‐related genes IL‐10, Arg1 and CD206 (Figure 4I). An addition of 1 µM ET_A_ inhibitor BQ123 or 1 µM ET_B_ inhibitor BQ788 to the culture significantly decreased the ET‐1‐induced elevation of YAP protein levels (Figure 4J,K). The data indicate that ET‐1‐driven microglial M1 phenotype is regulated by the activation of transcription coactivator YAP.

### 2.4 SCI‐Induced Thrombin Contributes to the Activation of Astrocytic ET‐1 System

**Figure 5:**
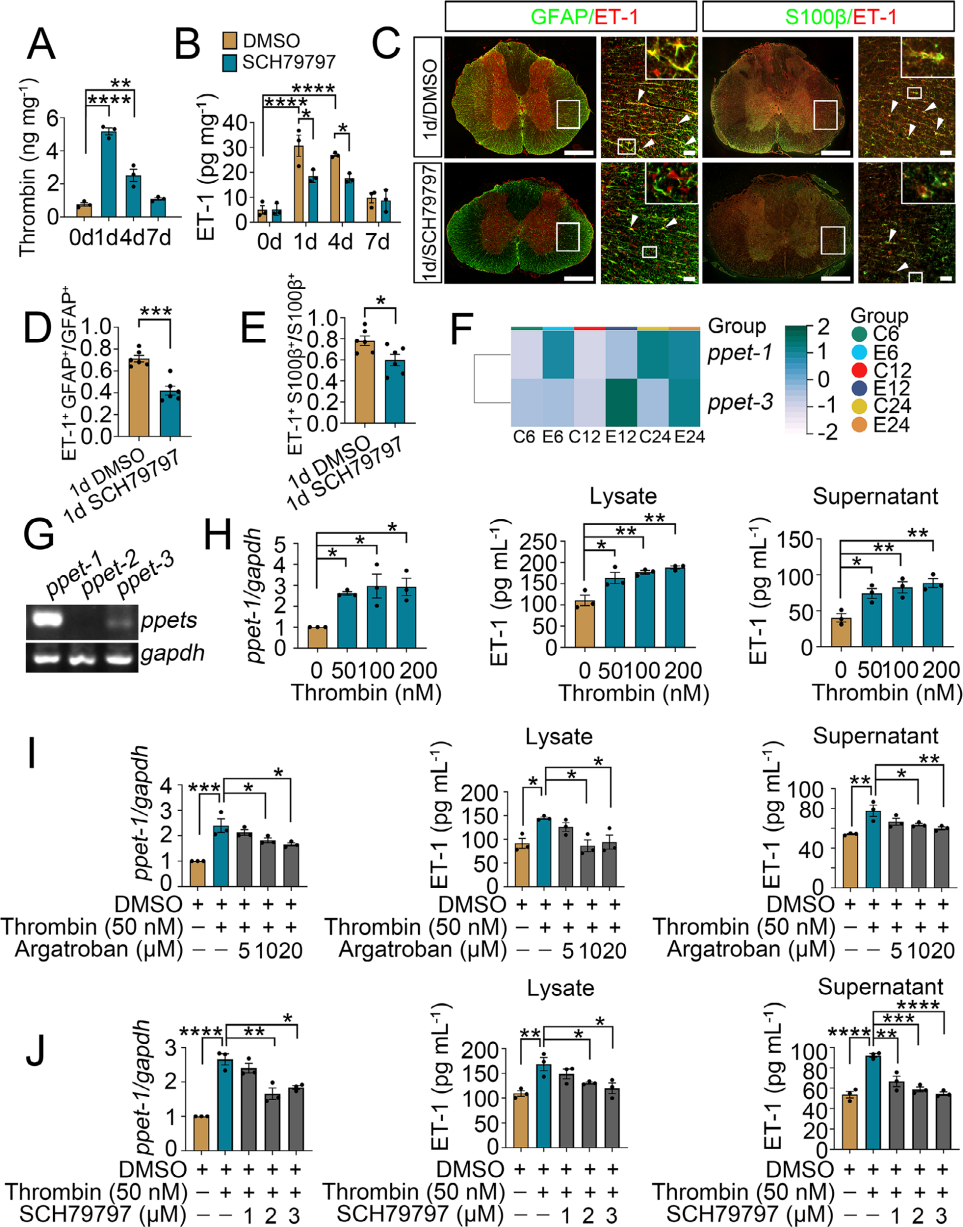
Determination of thrombin effects on astrocytic ET‐1 system activation following SCI. A) ELISA measurement of thrombin protein levels at 0 d, 1 d, 4 d and 7 d at lesion sites following SCI. Data are expressed as mean ± SEM, *n* = 3, ^**^ *p* < 0.01, ^****^ *p* < 0.0001, one­way analysis of variance followed by Dunnett's *post hoc* test. B) ELISA measurement of ET‐1 protein levels from spinal cord tissues following treatment with vehicle or 4.5 µL PAR‐1 inhibitor SCH79797 (50 µg kg^−1^) at lesion sites at 0 d, 1 d, 4 d and 7 d, respectively. The DMSO (0.1%) was used as vehicle. Data are expressed as mean ± SEM, n = 3, ^*^ *p* < 0.05, ^****^ *p* < 0.0001, two‐way analysis of variance followed by Sidak's *post hoc* test. C) Immunostaining of ET‐1 protein in the GFAP‐ and S100β‐positive astrocytes of injured spinal at 1 d following SCH79797 administration. Rectangle indicates the region magnified. Arrowheads indicate the positive signals. Scale bars, 500 µm and 50 µm in magnification. D, E) Quantification data as shown in (C). Data are expressed as mean ± SEM, *n* = 6, ^*^ *p* < 0.05, ^***^ *p* < 0.001, two‐tailed unpaired Student's *t*‐test. F) Expression analysis of the preproendothelins in the astrocytes by clustering heatmap following treatment of the cells with 100 nM thrombin for 6, 12 and 24 h, respectively. The color illustrates the relative expression levels of the preproendothelins. G) Semi‐quantitative PCR examined the expression levels of ppET‐1, ppET‐2 and ppET‐3 in primary astrocytes. Quantities were normalized to endogenous *gapdh*. H) qRT‐PCR and ELISA were used to determine the expression levels of ppET‐1 and ET‐1 following the astrocytes challenged with 0–200 nM thrombin for 12 h. Data are expressed as mean ± SEM, *n* = 3, ^*^ *p* < 0.05, ^**^ *p* < 0.01, one­way analysis of variance followed by Dunnett's *post hoc* test. I) qRT‐PCR and ELISA assay of the ppET‐1 and ET‐1 expression following the cells treatment with 5–20 µM thrombin inhibitor Argatroban in the presence of 50 nM thrombin for 12 h. Data are expressed as mean ± SEM, *n* = 3, ^*^ *p* < 0.05, ^**^ *p* < 0.01, ^***^ *p* < 0.001, one­way analysis of variance followed by Sidak's *post hoc* test. J) The expression of ppET‐1 and ET‐1 was determined using qRT‐PCR and ELISA after the cells treatment with 1–3 µM PAR‐1 inhibitor SCH79797 in the presence of 50 nM thrombin. Data are expressed as mean ± SEM, *n* = 3, ^*^ *p* < 0.05, ^**^ *p* < 0.01, ^***^ *p* < 0.001, ^****^ *p* < 0.0001, one­way analysis of variance followed by Sidak's *post hoc* test.

Now that the activation of astrocytic ET‐1 system determines M1 microglia phenotype at acute phase following SCI, an interest was evoked to clarify the key factors in regulating this system. Thrombin is able to induce intracellular formation of IP3 and mobilization of Ca^2+^, the upstream signaling necessary for ET‐1 production.^[^ ^7^, ^20^ ^]^ Therefore, the potential regulatory relations between them were interrogated. ELISA results displayed that the protein levels of thrombin at lesion site were significantly elevated at 1 d and 4 d following SCI (**Figure** **5**A), in parallel with those of ET‐1 (Figure 5B). As thrombin regulates intracellular signaling of glial cells mainly through activation of protease‐activated receptor‐1 (PAR‐1),^[^ ^19^, ^25^ ^]^ an intrathecal injection of 4.5 µL PAR‐1 inhibitor SCH79797 (50 µg kg^−1^) was thus allowed to investigate the changes of astrocytic ET‐1 production. The results showed that abrogation of thrombin signals markedly reduced the ET‐1 production at lesion site of the cord (Figure 5B). Immunostaining revealed that the abundance of ET‐1 in GFAP‐ and S100β‐positive astrocytes was accordingly decreased (Figure 5C‐E). The data indicate that SCI‐induced thrombin correlates with the activation of astrocytic ET‐1 system.

### 2.5 Thrombin Promotes Astrocytic Expression of ppET‐1 Through PAR‐1/NF‐κB Signaling

To examine the regulatory role of thrombin on astrocytic ET‐1 production, as well as the underlying mechanism, the primary astrocytes from the spinal cord were stimulated with 100 nM thrombin for 6, 12, and 24 h. Analysis of the differentially expressed genes (DEGs) available from previous transcriptome sequencing revealed that ppET‐1 and ppET‐3, rather than ppET‐2, were dynamically induced by the thrombin (Figure 5F). Semi‐quantitative PCR results showed that the ppET‐1 was the predominantly expressed subtype in the astrocytes in comparison with the ppET‐3 (Figure 5G; Figure S6A, Supporting Information). Exposure of the cells to 0–200 nM thrombin for 12 h showed that the expression of ppET‐1 was upregulated in a concentration‐dependent manner. Accordingly, the ET‐1 production was also elevated, as was determined by ELISA (Figure 5H). Addition of 5–20 µM thrombin inhibitor Argatroban in the presence of 50 nM thrombin, however, was found to abrogate the promoting effects of thrombin on the astrocytic expression of ppET‐1 and ET‐1 (Figure 5I). The data indicate that thrombin is efficient in facilitating astrocytic expression of ppET‐1.

To ascertain the exact receptor(s) for thrombin action on astrocytes, the cells were treated with PAR‐1 and PAR‐4 inhibitors or PAR‐3 siRNA. Treatment of astrocytes with 1–3 µM PAR‐1 inhibitor SCH79797 in the presence of 50 nM thrombin for 12 h, remarkably decreased the transcriptional level of the ppET‐1, and as a result, the astrocytic production of mature ET‐1 (Figure 5J). However, neither PAR‐3 siRNA (pre‐transfection for 48) nor 20–100 µM PAR‐4 inhibitor tcY‐NH_2_, exhibited similar inhibitory effects (Figure S6B–D, Supporting Information). The data indicate that PAR‐1 is the exclusive receptor of thrombin in activating astrocytic ppET‐1.

**Figure 6:**
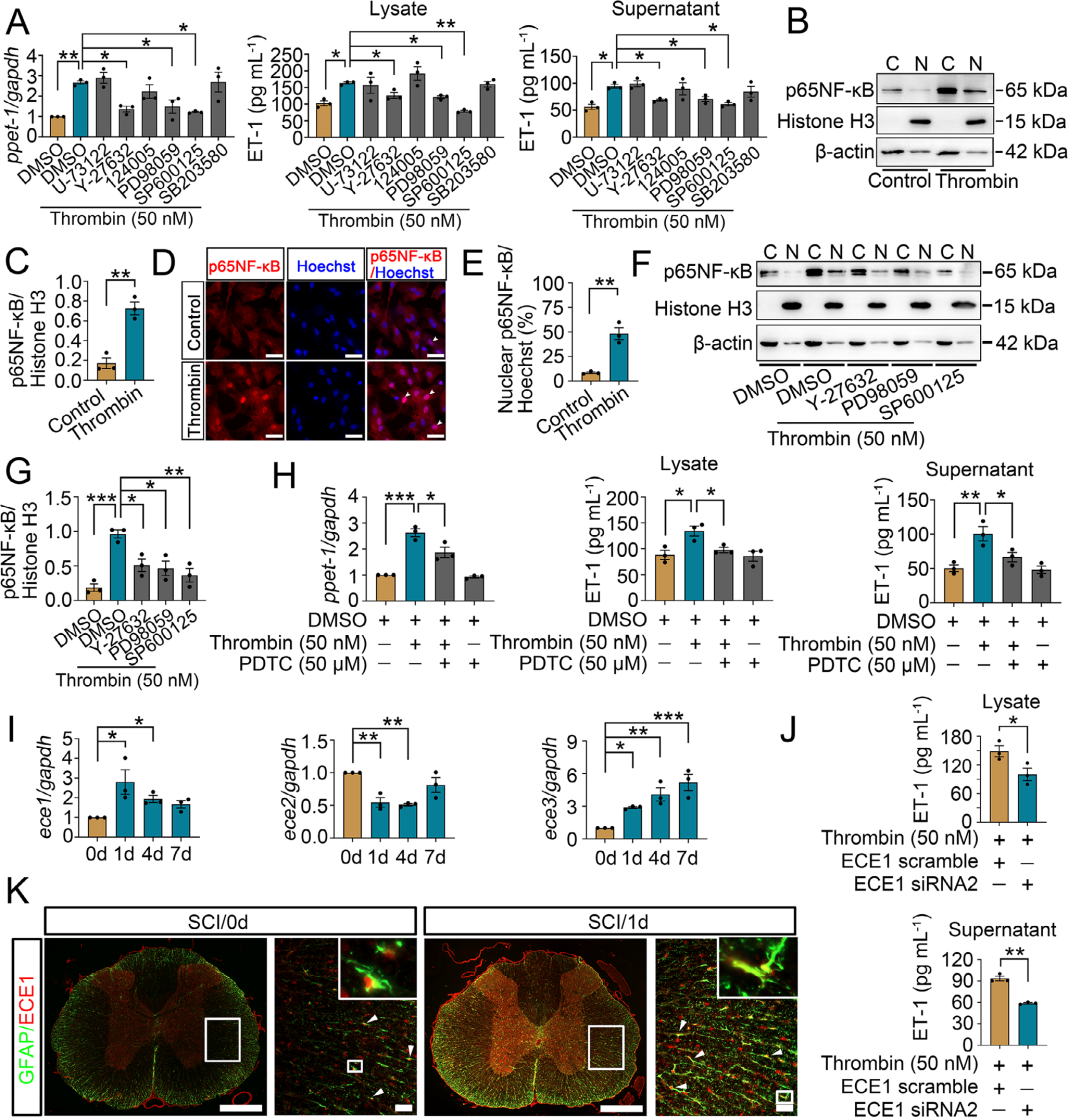
Signal pathway analysis of thrombin‐activated astrocytic ET‐1 system. A) qRT‐PCR and ELISA determination of ppET‐1 and ET‐1 expression in astrocytes after treatment with 1 µM PLCβ3 inhibitor U73122, 5 µM RhoA inhibitor Y‐27632, 20 µM AKT inhibitor 124 005, 10 µM ERK inhibitor PD98059, 10 µM JNK inhibitor SP600125 or 10 µM P38 inhibitor SB203580 in the presence of 50 nM thrombin for 12 h. Data are expressed as mean ± SEM, *n* = 3, ^*^ *p* < 0.05, ^**^ *p* < 0.01, one­way analysis of variance followed by Tukey's *post hoc* test. B) Western blot analysis of p65NF‐κB protein in the cytoplasm and nucleus following astrocytes treatment with 50 nM thrombin for 12 h. C) Quantification data as shown in (B). Data are expressed as mean ± SEM, *n* = 3, ^**^ *p* < 0.01, two‐tailed unpaired Student's *t*‐test. Quantities were normalized to endogenous β‐actin (cytoplasm) or Histone H3 (nucleus). D) Immunostaining showed the colocalization of p65NF‐κB with Hoechst 33 342 in the astrocytes treated by 50 nM thrombin for 12 h. Arrowheads indicate p65NF‐κB‐positive nucleus. Scale bars, 50 µm. E) Quantification data as shown in (D). Data are expressed as mean ± SEM, *n* = 3, ^**^ *p* < 0.01, two‐tailed unpaired Student's *t*‐test. F) Western blot analysis of p65NF‐κB protein in the cytoplasm and nucleus after astrocytes treatment with 5 µM RhoA inhibitor Y‐27632, 10 µM ERK inhibitor PD98059 or 10 µM JNK inhibitor SP600125 for 12 h in the presence of 50 nM thrombin. G). Quantification data as shown in (F). Data are expressed as mean ± SEM, *n* = 3, ^*^ *p* < 0.05, ^**^ *p* < 0.01, ^***^ *p* < 0.001, one­way analysis of variance followed by Sidak's *post hoc* test. Quantities were normalized to endogenous β‐actin (cytoplasm) or Histone H3 (nucleus). H) The expression of ppET‐1 and ET‐1 was determined by qRT‐PCR and ELISA following primary astrocytes challenged with 50 µM PDTC for 12 h in the presence of 50 nM thrombin, respectively. Data are expressed as mean ± SEM, *n* = 3, ^*^ *p* < 0.05, ^**^ *p* < 0.01, ^***^ *p* < 0.001, one­way analysis of variance followed by Sidak's *post hoc* test. I) qRT‐PCR examination of ECE1, ECE2 and ECE3 expression following rat SCI at 0 d, 1 d, 4 d and 7 d, respectively. Data are expressed as mean ± SEM, *n* = 3, ^*^ *p* < 0.05, ^**^ *p* < 0.01, ^***^ *p* < 0.001, one­way analysis of variance followed by Dunnett's *post hoc* test. Quantities were normalized to endogenous *gapdh*. J) ELISA measurement of the astrocytic ET‐1 production after knockdown of ECE1 expression for 48 h, followed by treatment with 50 nM thrombin for 12 h. Data are expressed as mean ± SEM, *n* = 3, ^*^ *p* < 0.05, ^**^ *p* < 0.01, two‐tailed unpaired Student's *t*‐test. K) Immunostaining showed colocalization of ECE1 with GFAP‐positive astrocytes before (0 d) and after SCI (1 d). Rectangle indicates the region magnified. Arrowheads indicate the positive signals. Scale bars, 500 µm and 50 µm in magnification.

As one of G protein‐coupled receptor (GPCR), PAR‐1 activation is involved in regulating downstream signals including PLCβ3, RhoA, AKT, and MAPKs, which converge to nuclear transcription factor NF‐κB to mediate multiple cellular responses.^[^ ^19^, ^26^ ^]^ To unveil the underlying mechanisms of thrombin‐induced ppET‐1 expression of astrocytes, the cells were treated with PLCβ3 (1 µM U‐73122), RhoA (5 µM Y‐27632), AKT (20 µM 124 005), ERK (10 µM PD98059), JNK (10 µM SP600125) or P38 inhibitor (10 µM SB203580) in the presence of 50 nM thrombin for 12 h. The results showed that inhibition of RhoA, ERK, or JNK activity significantly suppressed thrombin‐mediated promoting roles on astrocytic ppET‐1 expression and ET‐1 production (**Figure** **6**A). Next, the expression changes of p65NF‐κB were also examined. Treatment of the astrocytes with 50 nM thrombin for 12 h markedly increased the translocation of p65NF‐κB from cytoplasm to nucleus, as was determined by immunoblot for the cytosolic and nuclear fraction (Figure 6B,C) and immunostaining (Figure 6D and E). The inhibitor of RhoA (5 µM), ERK (10 µM), or JNK (10 µM) was found to attenuate thrombin‐induced p65NF‐κB elevation in the astrocytes (Figure 6F,G). In particular, an addition of 50 µM p65NF‐κB inhibitor PDTC was sufficient in restraining thrombin‐induced astrocytic expression of ppET‐1 and ET‐1 (Figure 6H). The data indicate that thrombin promotes astrocytic ppET‐1 expression through PAR‐1/RhoA/NF‐κB and PAR‐1/MAPKs/NF‐κB signal pathways.

### 2.6 Endothelin‐Converting Enzyme1 (ECE1) is Involved in Mature Peptide Processing of Thrombin‐Activated Astrocytic ET‐1 System

To date, a total of three isoforms of ECE, namely ECE1, ECE2, and ECE3 have been reported to be responsible for the final endothelins processing.^[^ ^7a^ ^]^ To identify the key ECEs responsible for astrocytic ET‐1 processing, we first examined the transcriptional levels of the three isoforms at lesion site of the cord. Results showed that the expression of ECE1 and ECE3, rather than ECE2, was markedly induced by SCI (Figure 6I). Next, the siRNAs targeting ECE1, ECE2, or ECE3 were designed to knock down the respective expression (Figure S7A, Supporting Information). Transfection of astrocytes with ECE1 siRNA2 for 48 h prior to stimulation with 50 nM thrombin for 12 h, significantly suppressed the production of ET‐1 (Figure 6J). However, neither ECE2 siRNA3 nor ECE3 siRNA2 caused remarkably inhibitory effects (Figure S7B,C, Supporting Information). Further determination by immunostaining of the cord sections displayed that the ECE1 colocalized with GFAP‐positive astrocytes before or after injury (Figure 6K). The data indicate that the ECE1 isoform is responsible for mature peptide processing of thrombin‐activated astrocytic ET‐1 system.

### 2.7 Thrombin‐Induced Astrocytic ET‐1 Mediates Microglia M1 Polarization

**Figure 7:**
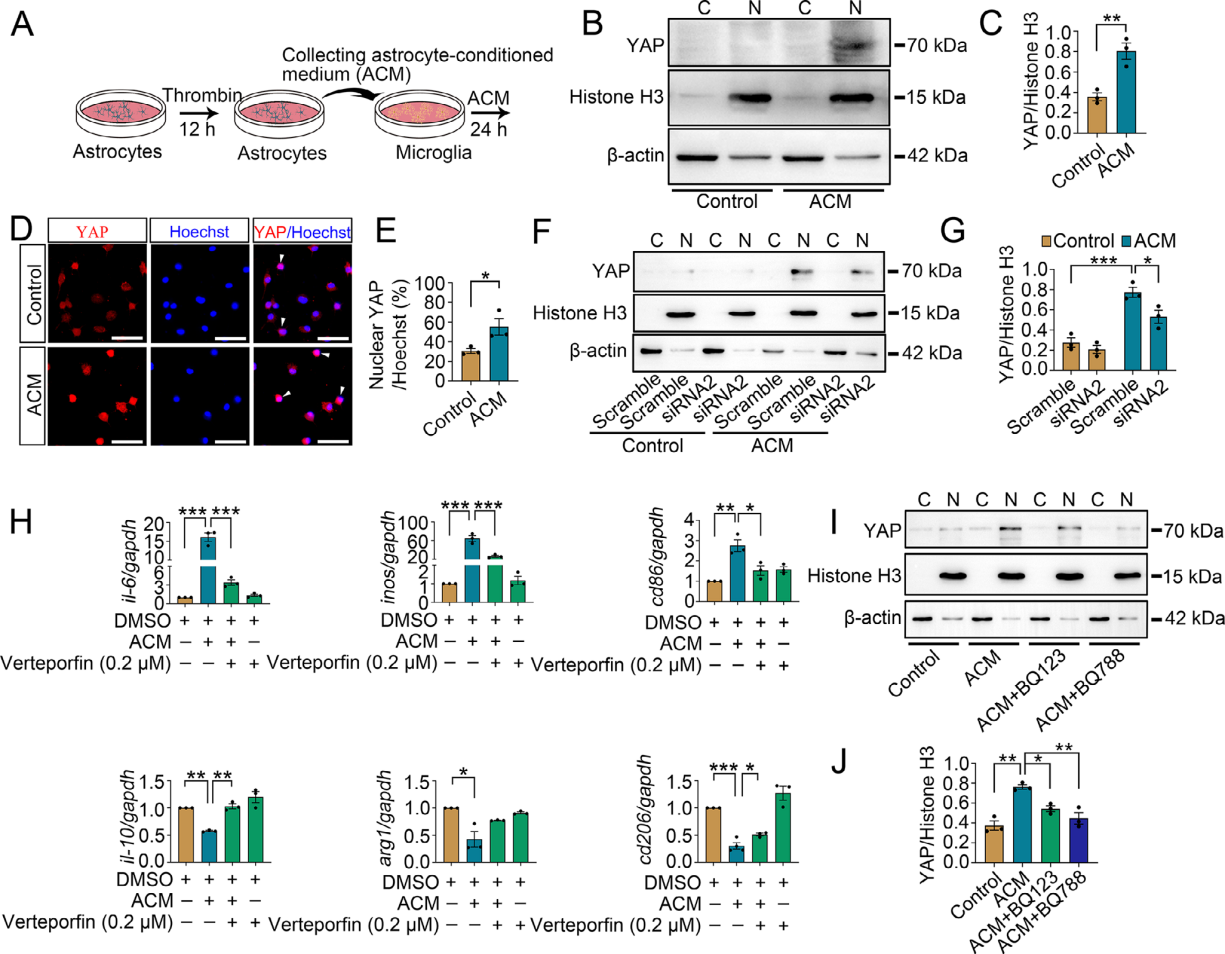
Effects of thrombin‐induced astrocyte‐conditioned medium (ACM) on microglial YAP activation and M1 polarization. A) Diagram of the ACM preparation. B) Western blot analysis of YAP protein levels in the cytoplasm and nucleus after treatment of microglia with ACM for 24 h, the ACM was prepared by stimulation of astrocytes with 50 nM thrombin for 12 h. C) Quantification data as shown in (B). Quantities were normalized to endogenous β‐actin (cytoplasm) or Histone H3 (nucleus). Data are expressed as mean ± SEM, *n* = 3, ^**^ *p* < 0.01, two‐tailed unpaired Student's *t*‐test. D) Immunostaining showed YAP translocation from cytoplasm to nucleus after treatment of the cells with ACM for 24 h. Arrowheads indicate YAP‐positive nucleus. The ACM was prepared by treatment of the astrocytes with 50 nM thrombin for 12 h. Scale bars, 50 µm. E) Quantifications of YAP subcellular localization with the nucleus co‐stained with Hoechst 33 342 as shown in (D). Data are expressed as mean ± SEM, *n* = 3, ^*^ *p* < 0.05, two‐tailed unpaired Student's *t*‐test. F) Western blot analysis of YAP protein levels in the cytoplasm and nucleus after stimulation of microglia with ACM for 24 h. The ACM was prepared by the interference of astrocytic ppET‐1 with siRNA2 for 48 h, followed by treatment with 50 nM thrombin for 12 h. G) Quantification data as shown in (F). Quantities were normalized to endogenous β‐actin (cytoplasm) or Histone H3 (nucleus). Data are expressed as mean ± SEM, *n* = 3, ^*^ *p* < 0.05, ^***^ *p* < 0.001, two‐way analysis of variance followed by Tukey's *post hoc* test. H) Determination of IL‐6, iNOS, CD86, IL‐10, Arg1 and CD206 following cell stimulation with ACM for 24 h in the presence of 0.2 µM YAP inhibitor Verteporfin. The ACM was prepared from astrocytes treated with 50 nM thrombin for 12 h. Data are expressed as mean ± SEM, *n* = 3‐4, ^*^ *p* < 0.05, ^**^ *p* < 0.01, ^***^ *p* < 0.001, one­way analysis of variance followed by Sidak's *post hoc* test. Quantities were normalized to endogenous *gapdh*. I) Western blot analysis of YAP protein levels in the cytoplasm and nucleus after treatment of microglia with ACM in the presence of 1 µM ET_A_ inhibitor BQ123 or 1 µM ET_B_ inhibitor BQ788 for 24 h. The ACM was prepared by treatment of astrocytes with 50 nM thrombin for 12 h. J) Quantification data as shown in (I). Data are expressed as mean ± SEM, *n* = 3, ^*^ *p* < 0.05, ^**^ *p* < 0.01, one­way analysis of variance followed by Sidak's *post hoc* test. Quantities were normalized to endogenous β‐actin (cytoplasm) or Histone H3 (nucleus).

To elucidate the role of thrombin‐induced astrocytic ET‐1 on the polarization of microglia, the primary astrocytes were transfected with or without ppET‐1 siRNA2 for 48 h prior to incubation with 50 nM thrombin for 12 h, followed by collection of the astrocyte‐conditioned medium (ACM). Stimulation of microglia with the ACM from ppET‐1 siRNA2 for 24 h remarkably decreased the expression of M1 phenotype‐related genes IL‐6, iNOS, and CD86, while increasing the M2 phenotype‐related genes IL‐10, Arg1 and CD206 compared with the scramble (**Figure** **7**A; Figure S8A–G, Supporting Information). An addition of 1 µM ET_A_ inhibitor BQ123 or 1 µM ET_B_ inhibitor BQ788 to the culture medium was able to reverse the ACM‐mediated alteration of those genes (Figure S8H, Supporting Information). The data indicate that thrombin‐induced astrocytic ET‐1 is indeed able to promote microglia M1 polarization through ET_A_ and ET_B_ receptors.

To verify the regulatory role of the YAP in ACM‐mediated microglia polarization, the YAP activation was determined by Western blot following microglia incubation at the ACM for 24 h. The results demonstrated that the ACM was efficient in promoting YAP translocation from cytoplasm to nucleus in microglia, as was shown by the cytosolic and nuclear fraction analysis and immunostaining (Figure 7B‐E). Meanwhile, the ACM prepared by the interference of astrocytic ppET‐1 with siRNA2 was able to suppress YAP activation of microglia (Figure 7F,G), suggesting the activating role of astrocytic ET‐1 on the YAP of microglia. Accordingly, an addition of 0.2 µM YAP inhibitor Verteporfin to the ACM‐stimulated microglia for 24 h restrained the ACM‐mediated promoting role on M1‐ and inhibitory role on M2‐related genes (Figure 7H). Microglia treatment with 1 µM ET_A_ inhibitor BQ123 or 1 µM ET_B_ inhibitor BQ788 in the presence of ACM for 24 h attenuated the activation of the YAP (Figure 7I,J). The data indicate that thrombin‐stimulated astrocytic ET‐1 regulates microglia M1 polarization by activating YAP signaling via ET_A_ and ET_B_ receptors.

### 2.8 Inhibition of Thrombin‐Activated Astrocytic ET‐1 System Favors for the Recovery of Rat Locomotor Function After SCI

**Figure 8:**
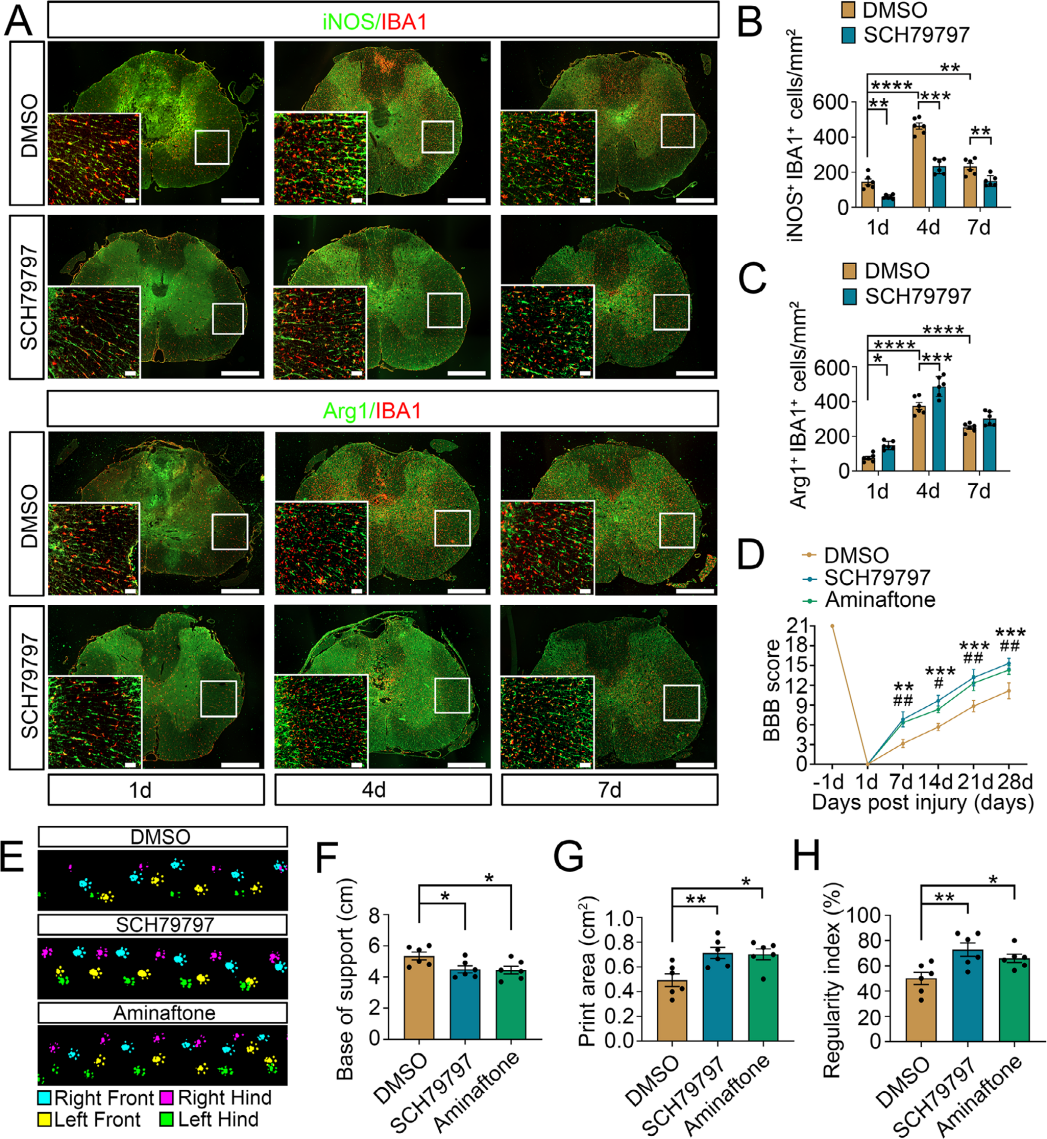
Pharmacological treatment with PAR‐1 or ET‐1 inhibitor improves the locomotor function of rats following SCI. A) Immunostaining of iNOS^+^ IBA1^+^ and Arg1^+^ IBA1^+^ microglia at the lesion site of the cord at 1 d, 4 d and 7 d following intrathecal injection of 4.5 µL PAR‐1 inhibitor SCH79797 (50 µg kg^−1^). The DMSO (0.1%) was used as vehicle. Rectangles indicate the region magnified. Scale bars, 500 µm and 50 µm in magnification. B, C) Quantification data as shown in (A). Data are expressed as mean ± SEM, *n* = 6, ^*^ *p* < 0.05, ^**^ *p* < 0.01, ^***^ *p* < 0.001, ^****^ *p* < 0.0001, two‐way analysis of variance followed by Sidak's *post hoc* test. D) Assessment of the hindlimb recovery of the subjects at 1 d, 7 d, 14 d, 21 d and 28 d by BBB score after administration of 4.5 µL PAR‐1 inhibitor SCH79797 (50 µg kg^−1^), ET‐1 inhibitor Aminaftone (500 µg kg^−1^) or DMSO (0.1%). Data are expressed as mean ± SEM, n = 6, ^**^ *p* < 0.01, ^***^ *p* < 0.001, (SCH79797 versus DMSO), ^#^ *P* < 0.05, ^##^ *P* < 0.01, (Aminaftone versus DMSO), two‐way analysis of variance followed by Tukey's *post hoc* test. (E) Footprint images of CatWalk gait at 21 d following intrathecal injection of various inhibitors. Cyan: right front; Magenta: right hind; Yellow: left front; Green: left hind. F‐H) CatWalk gait analysis of base of support (cm), print area (cm^2^) and regularity index (%) at 21 d after injury. Data are expressed as mean ± SEM. *n* = 6, ^*^ *p* < 0.05, ^**^ *p* < 0.01, one­way analysis of variance followed by Dunnett's *post hoc* test.

To assess the effects of thrombin‐activated astrocytic ET‐1 system on the functional outcomes of the damaged cord, the pharmacological approach was applied to treat the subjects immediately after the cord injury. An intrathecal injection of 4.5 µL PAR‐1 inhibitor SCH79797 (50 µg kg^−1^) demonstrated that inhibition of thrombin activity markedly reduced the number of iNOS^+^ and CD86^+^ microglia, while increasing the number of Arginase1^+^ and CD206^+^ microglia at lesion site at acute phase of SCI (**Figure** **8**A–C; Figure S9, Supporting Information). Behavioral tests of BBB score assessments showed that either SCH79797 or ET‐1 inhibitor Aminaftone (500 µg kg^−1^) was able to promote functional recovery of the rat hindlimb locomotor within 4 weeks (Figure 8D). Also, the base of support, print area, and regularity index were significantly improved at 21 d following SCI, as was shown by CatWalk gait analysis (Figure 8E‐H). The data indicate that inhibition of thrombin‐activated astrocytic ET‐1 system favors the recovery of rat locomotor function by reducing the number of M1 phenotype microglia after SCI.

## 3 Discussion

The endothelins system is a stress‐responsive regulator to carry out various physiological and pathological functions important in vascular biology, but also in development, cancer biology, and even neurobiology.^[^ ^7^, ^8^, ^15^ ^]^ Over the last three decades, investigation of the roles of ET‐1 in the vascular system has made significant progress, recapitulating its pathological effects on vasoconstriction, hypertension, atherosclerosis, cardiac disorders, pulmonary disease, and renal disease after being secreted from endothelial cells.^[^ ^7^, ^8^ ^]^ Recent evidence demonstrates that CNS disorders, such as ischemia, trauma, and neurodegenerative diseases, can result in obvious elevation of endothelins in the discrete areas to exacerbate neurological deficits.^[^ ^10^ ^]^ The expression of ET‐1 and/or its receptors ET_A_ and ET_B_ is detectable in glial and neuronal cells^[^ ^27^ ^]^ and in the nervous system vasculature,^[^ ^28^ ^]^ suggesting a wide array of functions including blood pressure, BBB permeability, respiratory control, and renal sympathetic neuronal activity.^[^ ^8^, ^29^ ^]^ In the present study, it was shown that the reactive astrocytes were the predominant cell types to inducibly produce ET‐1 in response to SCI, while other cells including endothelial cells, microglia and neurons presented undetectable changes in ET‐1 expression. These indicate that the astrocytic ET‐1 system is more susceptible to being activated than those of others including the vascular system, consistent with the findings in multiple sclerosis (MS).^[^ ^8b,c^ ^]^ Intriguingly, the astrocytes under physiological condition do not produce mature ET‐1. However, expression of ET‐1 and its receptors is strongly upregulated in the reactive astrocytes after CNS injury.^[^ ^10a^ ^]^ The released ET‐1 turns to act on these glial cells in a manner of autocrine, leading to cell proliferation, thereby forming a positive feedback in mediating the astrocytic ET‐1 production.^[^ ^10^, ^30^ ^]^ Such unique mode of astrocytic ET‐1 system activation implies the importance of targeting astrocytes in resolving CNS‐derived ET‐1 following insults. Notably, increased ET‐1 was also detected in the blood plasma immediately after SCI, suggesting a high diagnostic value for evaluating the activation of astrocytic ET‐1 system.

Microglia and macrophages are the key immune cells responsible for tissue surveillance and homeostasis in the CNS. As one of the first responders to the injury, the microglia/macrophages are mobilized within an hour and continue to accumulate for over a month.^[^ ^4c^ ^]^ During the acute and chronic phases of CNS injury, these cells play differential roles in the battle for neurological recovery by the acquisition of distinct phenotypes in response to various microenvironmental cues.^[^ ^31^ ^]^ Interestingly, the newly infiltrated microglia/macrophages at the lesion site express M2 phenotype‐related genes, whereas they are reversed to M1 phenotype and dominate the microenvironment about 1‐week following SCI.^[^ ^2^, ^4^ ^]^ The change in microglia M1/M2 ratio can result in a strong bias against CNS repairs because an increase in microglia M2 polarization has been found to alleviate secondary inflammatory‐mediated injury.^[^ ^2c^ ^]^ Although it is inadequate to oversimplify neurotoxicity of the M1 or neuroprotection of M2 phenotype of microglia/macrophages, the M1 phenotype is undoubtedly the source of destructive pro‐inflammatory mediators involved in expanding tissue damage.^[^ ^1^, ^32^ ^]^ In the present study, the activation of astrocytic ET‐1 system was shown as an early determinant of the microglia M1 phenotype following SCI. The findings not only interpret the mechanism of the predominant M1 phenotype at the acute phase of injury but provide alternative strategies for changing the dynamic process of the M1 microglia to improve neuroprotection.

ET‐1 acts through two seven transmembrane G‐protein coupled receptors ET_A_ and ET_B_ to exert its effects on pathophysiological process.^[^ ^33^ ^]^ Binding of ET‐1 to ET_A_ receptor will trigger an increase in intracellular calcium concentrations, leading to strong vasoconstriction and smooth muscle contraction.^[^ ^34^ ^]^ However, its interaction with ET_B_ receptor on endothelial cells can promote vasodilation by inducing the production of NO.^[^ ^34^ ^]^ ET‐1 was shown as the predominant neural ET after spinal cord injury, in consistency with those findings from previous studies,^[^ ^10^, ^35^ ^]^ suggesting the critical pathological role for ET‐1 in the ETs family in the damaged CNS. It appears that both ET_A_ and ET_B_ receptors are efficient in transmitting the ET‐1 signal, though the expression of ET_B_ receptor exceeds that of ET_A_ in the CNS.^[^ ^8^, ^36^ ^]^ The ET‐1 mediates various pathological functions through distinct signal pathways via the cell types‐specific ET receptors on the vascular smooth muscle cells, endothelial cells, radial glia, astrocytes, and epithelial tissues following CNS injury.^[^ ^10^, ^22^, ^35^ ^]^ For example, ET‐1 is able to induce reactive gliosis through ERK‐ and JNK‐dependent pathways via ET_B_ receptor.^[^ ^10^, ^37^ ^]^ While selective blockade of ET_A_ receptor is proven to be protective for stroke.^[^ ^38^ ^]^ As for microglia/macrophages, it was shown that the spinal cord microglial cells express both receptors with a high abundance of ET_B_, and all of them were inducibly upregulated following SCI. The selective pharmacological inhibitors of BQ123 and BQ788 were sufficient in suppressing the ET‐1‐mediated M1 polarization, suggesting the importance of blockade of ET_A_ and ET_B_ receptors at acute phase of SCI in controlling M1 phenotypic shift, as well as the resultant inflammatory activation.

Several transcription factors have been shown to regulate microglia/macrophage M1 polarization, including STAT1, IRF5, and IRF8.^[^ ^4^, ^24^, ^39^ ^]^ ET‐1 has been revealed to regulate transcription factors c‐FOS and c‐MYC via multiple signal pathways.^[^ ^7a^ ^]^ However, neither c‐FOS nor c‐MYC was efficient in the microglia M1 polarization. In various cell types, the ET‐1 mediates cell events in intracellular Ca^2+^‐dependent or ‐independent pathways.^[^ ^7^, ^40^ ^]^ As the elevation of intracellular Ca^2+^ is associated with subsequent cAMP accumulation,^[^ ^41^ ^]^ which in turn can bind to protein kinase A (PKA), resulting in phosphorylation of serine/threonine residues (p‐PKA) necessary for its optimal biological activity,^[^ ^42^ ^]^ the activity of YAP will thereafter be strongly inhibited by the p‐PKA.^[^ ^43^ ^]^ Herein, we elucidated that ET‐1 was able to promote microglia M1 polarization through activating YAP, suggesting an undefined regulatory mechanism of ET‐1 in Ca^2+^‐independent pathway.

Several soluble factors including TNF‐α, interleukins, insulin, norepinephrine, angiotensin II, and glucose have been shown to stimulate ET‐1 production in different cell types, especially the endothelial cells.^[^ ^7a^ ^]^ By promoting the ppET‐1 transcription, these factors are simultaneously able to activate the ECE family members responsible for ET‐1 processing. The reactive astrocytes have distinct signal transduction mechanisms for proinflammatory cytokines. For example, they are insufficient in activating HMGB1‐mediated inflammation due to lack of receptor‐interacting protein.^[^ ^44^ ^]^ As such, the critical activator of astrocytic ET‐1 system following SCI has not been clarified for a long time. The serine protease thrombin can enter the CNS through a disrupted BBB or blood‐spinal cord barrier (BSCB), and can also be released from neurons and glial cells in multiple types of CNS injury.^[^ ^19^, ^45^ ^]^ It promotes the expression of AP‐1, NF‐κB, and GATA‐2, which are essential for ppET‐1 transcription in a variety of cell types.^[^ ^19^, ^46^ ^]^ In addition, this serine protease is found to be active in upregulating ECE1 expression via Rho/ROCK and ERK pathway.^[^ ^47^ ^]^ In the present study, it was validated that thrombin acted as a key regulator in activating astrocytic ET‐1 system, suggesting a potential culprit for astrocytes dysfunction in the injured spinal cord. Likewise, thrombin has been extensively examined both in the vessel walls and senile plaques in AD,^[^ ^48^ ^]^ in plaques in MS,^[^ ^49^ ^]^ and in acute ischemic stroke,^[^ ^50^ ^]^ implying a broad pathological roles in astrocytes‐mediated degenerative diseases.

In conclusion, SCI‐induced thrombin activates the astrocytic ET‐1 system through RhoA/NF‐κB and MAPKs/NF‐κB signal pathway. The astrocytes‐derived ET‐1 in turn drives microglia M1 polarization at lesion site through regulation of YAP signaling via ET_A_ and ET_B_ receptors, thereby exacerbating pathological processes of the damaged spinal cord. The selective pharmacological inhibitors of PAR‐1 and ET‐1 are highly efficient in improvement of the rat locomotor function after SCI.

## 4 Experimental Section

### Animals

Adult male Sprague‐Dawley (SD) rats, weighing 180–220 g, were obtained from the Center of Experimental Animals at Nantong University. All animal experiments were approved by *the Animal Care and Use Committee of Nantong University* and the *Animal Care Ethics Committee of Jiangsu Province* (License No. SYXK (Su) 2020‐0029). All subjects had free access to food and water and were kept in standard cages with five rats per cage in an air‐conditioned room with a temperature of 22 ± 2 °C on a 12‐12 h light‐dark cycle.

### Establishment of Rat Sci Model and Pharmacological Treatment

The number of animals subjected to surgery was calculated by six per experimental group in triplicate. The rat spinal cord contusion model was established according to the previous description.^[^ ^51^ ^]^ In a nutshell, all subjects were anesthetized by an intraperitoneal injection of sodium pentobarbital (35 mg kg^−1^, Toronto Research Chemicals). The fur around the surgical site was shaved and the skin was disinfected with iodophor. Then the spinous processes of T8‐T10 vertebrae were surgically exposed, and a laminectomy was performed at the ninth thoracic vertebral level (T9) with the dura remaining intact. The exposed cord segment was maintained for stability, avoiding any displacement or movement before receiving a 150‐kilodyne contusion injury using the IH‐0400 Impactor (Precision Systems and Instrumentation) injury device. The impact rod was removed immediately, and edema and/or bleeding were subsequently observed before the incisions were cleaned and sutured. For drug delivery, 4.5 µL PAR‐1 inhibitor SCH79797 (50 µg kg^−1^, R&D systems, Cat#1592) or ET‐1 inhibitor Aminaftone (500 µg kg^−1^, TargetMol, Cat# T14219) were intrathecally injected at the lesion site before the incision suture. The control group received 0.1% DMSO (MilliporeSigma, Cat# D2650) at the same dose. The subjects were subcutaneously administered with 0.2 mL antibiotics, and manual expression of bladders was performed twice a day until the subjects recovered spontaneous voiding.

### Construction and Administration of the Adeno‐Associated Virus

A recombinant adeno‐associated virus type 5 (AAV5) system was commercially designed by Cyagen Biotechnology Co., Ltd. (Guangzhou, China). In brief, a DNA fragment encoding short hairpin RNA (shRNA) for targeting rat ppET‐1was inserted into AAV5 vector with mCherry fluorescent labels. The expression of AAV5 vector was driven by GFAP promoters (AAV5‐GFAP‐mCherry‐ppET‐1‐shRNA), resulting in astrocyte‐specific knockdown of ppET‐1. The ppET‐1 target sequences: 5′‐CCG AGC ACA TTG ACT ACA GAG C‐3′. Scrambled sequences 5′‐ACT AAG GTT AAG TCG CCC TCG‐3′ were used as controls, For AAV5 transfection, the rats were anesthetized and a laminectomy was performed as above, a total of 10 µL AAV5‐GFAP‐mCherry‐ppET‐1‐shRNA (1 × 10^13^ vg mL^−1^) or AAV5‐GFAP‐mCherry‐Scramble were injected at the surgical site, and were allowed for viral expression for 21 d before further experiments.

### Cell Culture and Treatment

Primary astrocytes and microglia were isolated from the spinal cord of newborn SD rats at 1–2 days after birth according to the previous description.^[^ ^51^ ^]^ Briefly, the dissected cords were immersed into 0.01 M PBS containing 1% penicillin‐streptomycin (Beyotime, Cat# C0222), followed by mincing with scissors and digestion with 0.25% trypsin (Gibco, Cat# 25200072) for 15 min at 37 °C. The digestion was terminated by adding Dulbecco's Modified Eagle's Medium (DMEM) – high glucose medium (MilliporeSigma, Cat# D6546) containing 10% fetal bovine serum (FBS, Gibco, Cat# 16 140 071), 1% penicillin‐streptomycin and 1% L‐glutamine (Beyotime, Cat# C0212). The suspension was then centrifuged at 1200 rpm for 5 min, and the cells were resuspended and seeded onto poly‐L‐lysine (MilliporeSigma, Cat# P4707) pre‐coated culture flask in the presence of 5% CO_2_. The medium was changed every 3 days until the whole flask was covered with cells. After 7–9 days, the culture flask was shaken at 250 rpm for 12 h, and the bottom of cells was collected to culture astrocytes. Alternatively, the culture flask was shaken at 180 rpm for 30 min and the supernatant was collected to culture the microglia. The phenotype of astrocytes and microglia was evaluated by a characteristic morphology and positive staining of GFAP (1:400, MilliporeSigma, Cat# G3893) or IBA1 (1:200, Wako, Cat# 019‐19741). The astrocytes or microglia with a purity of more than 95% were acceptable for subsequent experiments.

To examine the effects of thrombin‐mediated astrocytic ET‐1 on the microglia polarization as well as their underlying mechanisms, the microglia were planted in six‐well plates (1 × 10^5^ cells/well) and cultured for 24 h. After removing the culture medium, the cells were washed three times with 0.01 M PBS, followed by stimulation with 0–200 ng mL^−1^ recombinant rat ET‐1 (biomatik, Cat# RPC28542), or astrocyte‐conditioned medium (ACM), in the presence or absence of selective inhibitor BQ123 (for ET_A_, TOCRIS, Cat# 1188), BQ788 (for ET_B_, TOCRIS, Cat# 1500), or Verteporfin (for YAP, MCE, Cat# HY‐B0146) for 24 h prior to assay.

To examine the effects of thrombin on the astrocytic expression of ET‐1 and related mechanism, the astrocytes were planted in six‐well plates (2 × 10^5^ cells/well) and cultured for 24 h. After removing the culture medium, the cells were washed three times with 0.01 M PBS, followed by stimulation with 0–200 nM rat thrombin (MilliporeSigma, Cat# T5772) in serum‐free DMEM – high glucose medium. In addition, the cells were treated with 50 nM thrombin in the presence or absence of selective inhibitor Argatroban (for thrombin, MilliporeSigma, Cat# 1 042 408), SCH79797 (for PAR‐1, R&D systems, Cat# 1592), tcY‐NH_2_ (for PAR‐4, TOCRIS, Cat# 1488), PDTC (for NF‐κB, Beyotime, Cat# S1809), SB203580 (for P38, TOCRIS, Cat# 1202), SP600125 (for JNK, TOCRIS, Cat# 1496), PD98059 (for ERK, TOCRIS, Cat# 1213), Y‐27632 (for Rho‑kinase, MilliporeSigma, Cat# Y0503), U‐73122 (for PLC, MilliporeSigma, Cat# U6756) or 124 005 (for AKT, MilliporeSigma, Cat# 124005) for 12 h prior to assay.

For knockdown the expression of PAR‐3, ECEs or ET‐1 in the astrocytes, the cells were cultured on the six‐well plates (1.5 × 10^5^ cells/well) for 24 h, followed by transfection of PAR‐3 siRNA (sense strand 5′‐CCA ACA UCA UAC UCA UAA U dTdT‐3′, antisense strand 5′‐A UUA UGA GUA UGA UGU UGG dTdT‐3′), ET‐1 siRNA2 (sense strand 5′‐CGA GCA CAU UGA CUA CAG A dTdT‐3′, antisense strand 5′‐U CUG UAG UCA AUG UGC UCG dTdT‐3′), ECE1 siRNA2 (sense strand 5′‐CAU CAA CAG CAC CGA CAA A dTdT‐3′, antisense strand 5′‐U UUG UCG GUG CUG UUG AUG dTdT‐3′), ECE2 siRNA3 (sense strand 5′‐CUG AAC CUA UAC AAC UUC U dTdT‐3′, antisense strand 5′‐A GAA GUU GUA UAG GUU CAG dTdT‐3′) ECE3 siRNA2 (sense strand 5′‐GAC UCA GAA ACU ACG GAA A dTdT‐3′, antisense strand 5′‐GAC UCA GAA ACU ACG GAA A dTdT‐3′) or scramble siRNA (sense strand 5′‐GGC UCU AGA AAA GCC UAU GC dTdT‐3′, antisense strand 5′‐GC AUA GGC UUU UCU AGA GCC dTdT‐3′) with iMAX transfection reagent (Invitrogen, Cat# 13778150) for 24 h. The astrocytes were subsequently incubated at DMEM – high glucose medium for 24 h before being harvested for Q‐PCR or stimulated by 50 nM thrombin for ELISA assay.

### Western Blot

Protein was extracted from cells with a buffer containing 50 mM Tris (pH 7.4), 150 mM NaCl, 1% Triton X‐100, 1% sodium deoxycholate, 0.1% SDS and 1 mM PMSF following astrocytes treatment with thrombin for 12 h, or normal cultured microglia, or microglia stimulated with ET‐1 recombinant protein (200 ng mL^−1^) or astrocyte‐conditioned medium (ACM) for 24 h in the presence or absence of selective inhibitor. Samples were vortexed for 30 min and centrifuged at 12000 rpm for 15 min. The supernatants were collected and stored at −20 °C prior to assay. The protein concentration of each sample was detected by the Bradford method (Beyotime, Cat# P0006) to maintain the same loads according to the manufacturer's instructions. Proteins were heated at 95 °C for 5 min, and 20 µg of each sample was electrophoretically separated on 10% SDS‐PAGE gel (Beyotime, Cat# P0012AC), followed by transferring onto a polyvinylidene difluoride (PVDF) membrane (BioRad). The membrane was blocked with 5% skim milk (Beyotime, Cat# P0216) in Tris‐buffered saline containing 0.1% Tween‐20 for 1 h, and then incubated with primary antibody diluted with 0.01 M PBS at 4 °C for 24 h. The primary antibodies were used as follows: rabbit anti‐p65NF‐κB (1:1000, CST, Cat# 8242S); rabbit anti‐YAP (1:1000, CST, Cat# 14074S); rabbit anti‐c‐FOS (1:1000, CST, Cat# 2250S); rabbit anti‐c‐MYC (1:1000, Affinity, Cat# AF6054); rabbit anti‐ET_A_ (1:1000, Alomone Labs, Cat# AER‐001); rabbit anti‐ET_B_ (1:1000, Alomone Labs, Cat# AER‐002); β‐actin (1:5000, Proteintech, Cat# 66009‐1‐Ig). After washing 3 times with TBST for 10 min each, the membrane was incubated with secondary antibody goat‐anti‐mouse HRP (1:1000, Beyotime, Cat# A0216) or goat‐anti‐rabbit HRP (1:1000, Beyotime, Cat# A0208) for 2 h at room temperature. The HRP activity was detected using an enhanced chemiluminescence (ECL) kit (Beyotime, Cat# P0018). The image was scanned with a GS800 Densitometer Scanner (Bio‐Rad), and the data were analyzed using PDQuest 7.2.0 software (Bio‐Rad).

### Subcellular Fractionation

The microglia were stimulated with ET‐1 recombinant protein (200 ng mL^−1^) or the ACM in the presence of ET_A_ inhibitor BQ123 (1 µM) or ET_B_ inhibitor BQ788 (1 µM) for 24 h, while the astrocytes were stimulated with thrombin (50 nM) in the presence of RhoA inhibitor Y‐27632 (5 µM), ERK inhibitor PD98059 (10 µM) or JNK inhibitor SP600125 (10 µM) for 12 h. Extraction of nuclear and cytosolic protein from the cell was carried out according to the procedure of the Nuclear and Cytoplasmic Protein Extraction Kit (Beyotime, Cat# P0028). Briefly, the cells were washed with ice‐cold PBS, followed by lysis in 200 µL cytoplasmic protein extraction agent A, supplemented with 1 mM PMSF on ice for 15 min before a vortex for 5 s. Then, the cytoplasmic protein extraction agent B was added, and the mixture was vortexed for 5 s prior to incubation on ice for 1 min. Samples were centrifuged at 16 000 g at 4 °C for 5 min, and the supernatant (cytosolic fraction) was immediately collected. The pellet (nuclear fraction) was resuspended in nuclear protein extraction agent supplemented with 1 mM PMSF. After 15 to 20 times vortex for 30 min, centrifugation was performed at 16 000 g for 10 min. The supernatants containing the nuclear extracts were thus collected. Proteins of nuclear and cytosolic extracts were determined by Western blot. The β‐actin or Histone H3 (1:1000, CST, Cat# 9715S) were used as the internal control of cytoplasmic or nuclear proteins, respectively.

### Enzyme Linked Immunosorbent Assay (ELISA)

Cells or tissues were sonicated using the lysis buffer supplemented with a protease inhibitor PMSF as mentioned above. Homogenate was centrifuged at 12 000 rpm for 15 min at 4 °C, and the supernatant was collected for ET‐1 ELISA assay (R&D systems, Cat# DET100). The blood plasma of rat was prepared from the tail veins with a vacuum blood collection system using EDTA as an anticoagulant before centrifugation at 4600 rpm for 30 min at 4 °C. The supernatant was used for ET‐1 assay. The concentration of ET‐1 was expressed as pg mL^−1^ for the supernatant and lysate of the cells, while the pg mg^−1^ for the cord tissues. Plates were read with a multifunctional enzyme marker (Biotek Synergy2) at a 450 nm wavelength.

### Cell Viability Assay

The microglia were planted in 96‐well plates with 6 × 10^3^ cells/well and cultured for 24 h. The cells were treated with different concentrations of BQ123 (TOCRIS, Cat# 1188), BQ788 (TOCRIS, Cat# 1500), 10058‐F4 (MCE, Cat# HY‐12702) or Verteporfin (MCE, Cat# HY‐B0146) for 24 h. After addition of 100 µL of 3‐(4,5‐dimethyl‐2‐thiazolyl)‐2,5‐diphenyl‐2‐H‐tetrazolium bromide (0.5 mg mL^−1^, MTT, Sangon, Cat# A600799) for 4 h, the DMSO (MilliporeSigma, Cat# D2650) was added to dissolve formazan for 10 min in the dark. The cell viability was evaluated by detecting the absorbance at 570 nm with a Microplate Reader (BioTek, Synergy2).

### Quantitative Polymerase Chain Reaction (Q‐PCR)

The transcripts were measured with real‐time polymerase chain reaction (qRT‐PCR) and quantified by the 2^−ΔΔCT^ method. In brief, total RNA was prepared with Trizol (MilliporeSigma, Cat# T9424) from astrocytes following different treatment. The first‐strand cDNA was synthesized using HiScript II Q RT SuperMix for qPCR (+gDNA wiper) (Vazyme, Cat# R223) in a 20 µL reaction system, and diluted at 1:3 before assay. The specific primers shown in Table S1 (Supporting Information) were designed and synthesized by Invitrogen (Shanghai, China). Reactions were performed in a final volume of 10 µL (1 µL cDNA template and 9 µL reaction buffer containing 5 µL of 2 × ChamQ Universal SYBR qPCR Master Mix (Vazyme, Cat# Q711), 3 µL of RNase free ddH_2_O, and 0.5 µL of anti‐sense and sense primers each). Reactions were processed using one initial denaturation cycle at 94 °C for 5 min, followed by 40 cycles of 94 °C for 30 s, 60 °C for 30 s, and 72 °C for 30 s. Fluorescence was recorded during each annealing step. At the end of each PCR run, data were automatically analyzed by the system, and amplification plots were obtained. The expression levels were normalized to the endogenous *gapdh*. In addition, a negative control without the first‐strand cDNA was also performed.

### Immunostaining

Spinal cord segments were harvested from six experimental subjects at each time point after SCI and drug administration, post‐fixed in 4% paraformaldehyde at 4 °C for 4 h. Then, the tissues were dehydrated by 10%, 20%, and 30% gradient sucrose (in 0.01 M PBS) for 24 h, respectively. The segments were embedded in Tissue‐Tek Optimal Cutting Temperature (OCT) compound (VWR International), and were sectioned at 12 µm in a cryostat from 0.25 cm length to the lesion epicenter. Three sections of each sample surrounding the area of injury were chosen for the immunostaining. For cell preparation, the microglia were cultured and fixed with 4% paraformaldehyde for 45 min after stimulation with ET‐1 recombinant protein (200 ng mL^−1^) or ACM for 24 h. Both sections and the cells were blocked with 0.01 M PBS containing 3% BSA (BioFroxx, Cat# 4240GR100), 0.1% Triton X‐100 (Beyotime, Cat# ST797), and 10% normal goat serum (Bioss, Cat# C‐0005) for 1 h at 37 °C, and incubated with primary antibody for 24 h at 4 °C. The primary antibodies used for immunostaining were as follows: rabbit anti‐ET‐1 (1:200, Abcam, Cat# ab117757); mouse anti‐GFAP (1:400); mouse anti‐S100β (1:400, Abcam, Cat# ab218515); mouse anti‐NeuN (1:400, Abcam, Cat# ab104224); mouse anti‐OX42 (1:200, Abcam, Cat# ab1211); goat anti‐CD31 (1:200, Novus, Cat# AF3628); rabbit anti‐ECE1 (1:200, proteintech, Cat# 26088‐1‐AP); rabbit anti‐ET_A_ (1:100, Alomone Labs, Cat# AER‐001); rabbit anti‐ET_B_ (1:100, Alomone Labs, Cat# AER‐002); mouse anti‐CD86 (1:100, Abcam, Cat# ab270719); mouse anti‐CD206 (1:100, Abcam, Cat# ab8918); mouse anti‐Arg1 (1:100, proteintech, Cat# 66129‐1‐Ig); mouse anti‐iNOS (1:200, abcam, Cat# ab49999); rabbit anti‐IBA1 and rabbit anti‐YAP (1:200, CST, Cat# 14074S). The sections were subsequently rinsed with PBS and incubated with the Cy3‐labeled goat anti‐rabbit IgG (1:400, Abcam, Cat# ab6939), the Alexa Fluor 488‐labeled donkey anti‐mouse IgG (1:400, Abcam, Cat# ab150109), the Alexa Fluor 647‐labeled donkey anti‐rabbit IgG (1:400, Abcam, Cat# ab150075), or the Alexa Fluor 488‐labeled donkey anti‐goat IgG (1:400, Abcam, Cat# ab150129) for 24 h at 4 °C. Immunostainings were observed under a fluorescence microscope (ZEISS, axio image M2). Quantification of iNOS^+^ IBA1^+^, CD86^+^ IBA1^+^, Arginase1^+^ IBA1^+,^ and CD206^+^ IBA1^+^ cells in the spinal cord tissues, or the positive signals of YAP in the microglia was analyzed by Image‐Pro Plus 6.0 software (Media Cybernetics, Rockville, Md, USA). Colocalization analysis of signals was carried out using the colocalization plugin (Colocalization Finder) on Image‐Pro Plus as previous description.^[^ ^52^ ^]^ Three sections of each cord and 15 fields in each section were statistically analyzed.

### Bioinformatics Analysis of Transcriptome

The heatmap of differentially expressed genes (DEGs), which were obtained from previous data,^[^ ^19c^ ^]^ was clustered by Jensen–Shannon divergence and was drawn by the ggplot2 in the R language packages.

### Behavioral Tests

The hindlimb locomotor function was assessed by open‐field test and CatWalk analysis as previously described.^[^ ^53^ ^]^ The standardized Basso, Beattie, and Bresnahan (BBB) score was evaluated using the Open‐field test at 1, 7, 14, 21, and 28 days. Three well‐trained investigators blind to the experiments were invited to observe the behavior of subjects for 5 min. The BBB scores ranged from 0 to 21 according to the rating scale, with a score of 21 before surgery and 0 to 1 after a successful SCI. The locomotor gaits of the subjects were quantitatively assessed by the CatWalk automated gait analysis system (Noldus Information Technology, the Netherlands). The subjects were pre‐trained across the smooth glass runway for 3 days before injury. At least three uninterrupted crossings were effectively obtained for each subject during the tests. Footprints for each animal were manually marked and CatWalk software (Version 10.6) automatically calculated the gait parameters. The parameters including the base of support, print area, and regularity index were used to assess hindlimb function. All the parameters were averaged for the left and right hindlimb per subject.

### Statistical Analysis

GraphPad Prism 8 software (San Diego, CA, USA) was used for statistical analysis. All data were presented as mean ± standard error of mean (M ± SEM). Comparisons between two groups following a normal distribution were analyzed by two‐tailed unpaired Student's *t*‐test. Differences between multiple groups were analyzed by one­way analysis of variance (ANOVA) or two‐way ANOVA, followed by Dunnett's, Tukey's, or Sidak's *post hoc* test. Single, double, triple, and quadruple asterisks represent *P* < 0.05, 0.01, 0.001, and 0.0001, respectively. *P* value < 0.05 was considered statistically significant.

## Conflict of Interest

The authors declare no conflict of interest.

## Author Contributions

Yjun W. and B.H. contributed to conceptualization, methodology, and software. Yjun W. was responsible for original draft preparation, revision, and supervision. Validation and funding acquisition were carried out by Yjun W., Yjie W., Z.C., and B.H. Data curation was performed by Yjun W., B.H., S.X., M.L., H.L., S.L., L.N., H.S., Y.Z., and Yjie W., while visualization and investigation were conducted by R.C., Y.Z., Z.C., and Yjie W. All authors have reviewed and approved the current version of the manuscript and agree to be accountable for all aspects of the work concerning its accuracy and integrity.

## Ethical Statement

All animal experiments were approved by the Animal Care and Use Committee of Nantong University and the Jiangsu Province Animal Care Ethics Committee (License No. SYXK (Su) 2020‐0029).

## Supporting information



Supporting Information

Supporting Information

## Data Availability

The data that support the findings of this study are available from the corresponding author upon reasonable request.
