# Supplementary material for: Astrocytic ET‐1 System Determines Microglia Phenotype Following Spinal Cord Injury
Source: Adv Sci (Weinh). 2025 May 30;12(31):e07215. doi: 10.1002/advs.202507215 (PMC12376558; doi:10.1002/advs.202507215)
Supplement: Supplementary file 1 — Supporting Information [file ADVS-12-e07215-s002.doc]

**Astrocytic ET-1 System Determines Microglia Phenotype Following Spinal Cord Injury**

*Bingqiang He*, *Si Xu*, *Mengdi Li*, *Hui Li*, *Shaolan Li*, *Li Niu*, *Honghua Song*, *Rixin Cai*, *Yue Zhou*, *Zhilong Cao*, *Yingjie Wang*, *Yongjun Wang**

B. He, S. Xu, M. Li, H. Li, S. Li, L. Niu, H. Song, R. Cai, Y. Zhou, Z. Cao, Y. Wang, Yjun. Wang

Key Laboratory of Neuroregeneration of Jiangsu and Ministry of Education, Co-innovation Center of Neuroregeneration

Nantong University

19 Qixiu Road, Nantong 226001, PR China

Email: wyjbs@ntu.edu.cn

B. He, H. Li, H. Song, R. Cai, Y. Zhou, Z. Cao

Medical School of Nantong University

19 Qixiu Road, Nantong 226001, PR China

Yjun. Wang

Department of Burn and Plastic Surgery

Affiliated Hospital of Nantong University

20 Xisi Road, Nantong, 226001, PR China

**
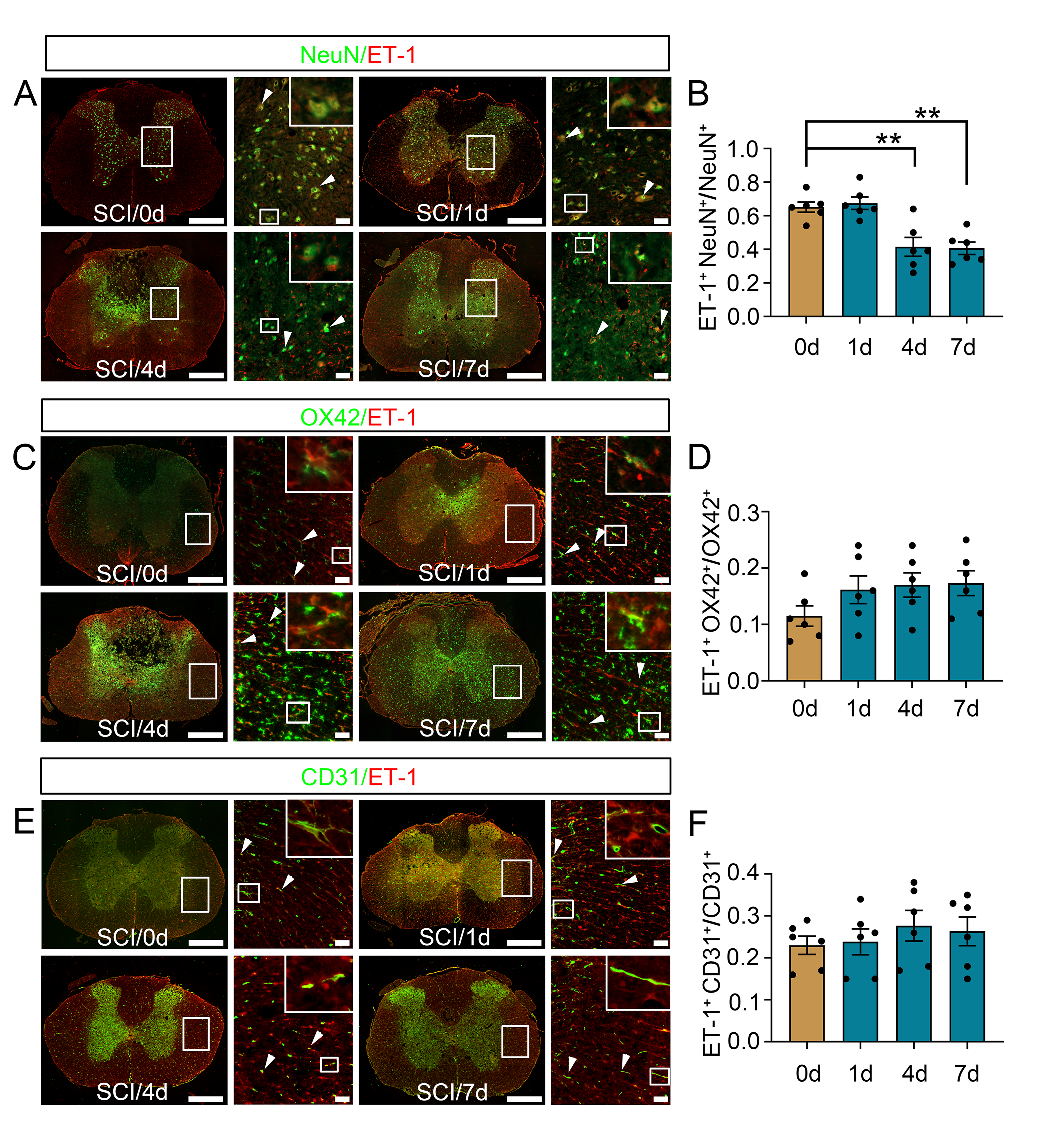
**

**Figure S1.** Analysis of ET-1 expression changes within neurons, microglia and endothelial cells following rat SCI. A, C and E) Immunostaining showed colocalization of ET-1 with NeuN-positive neurons (A), OX42-positive microglia (C) or CD31-positive endothelial cells (E) at 0 d, 1 d, 4 d and 7 d following SCI. Rectangles indicate the region magnified. Arrowheads indicate the positive signals. Scale bars, 500 μm in (A), (C) and (E); 50 μm in magnification. B, D and F) Quantification data as shown in (A), (C) and (E), respectively. Data are expressed as mean ± SEM, *n* = 6, ***P* < 0.01, one­way analysis of variance followed by Dunnett's *post hoc* test.


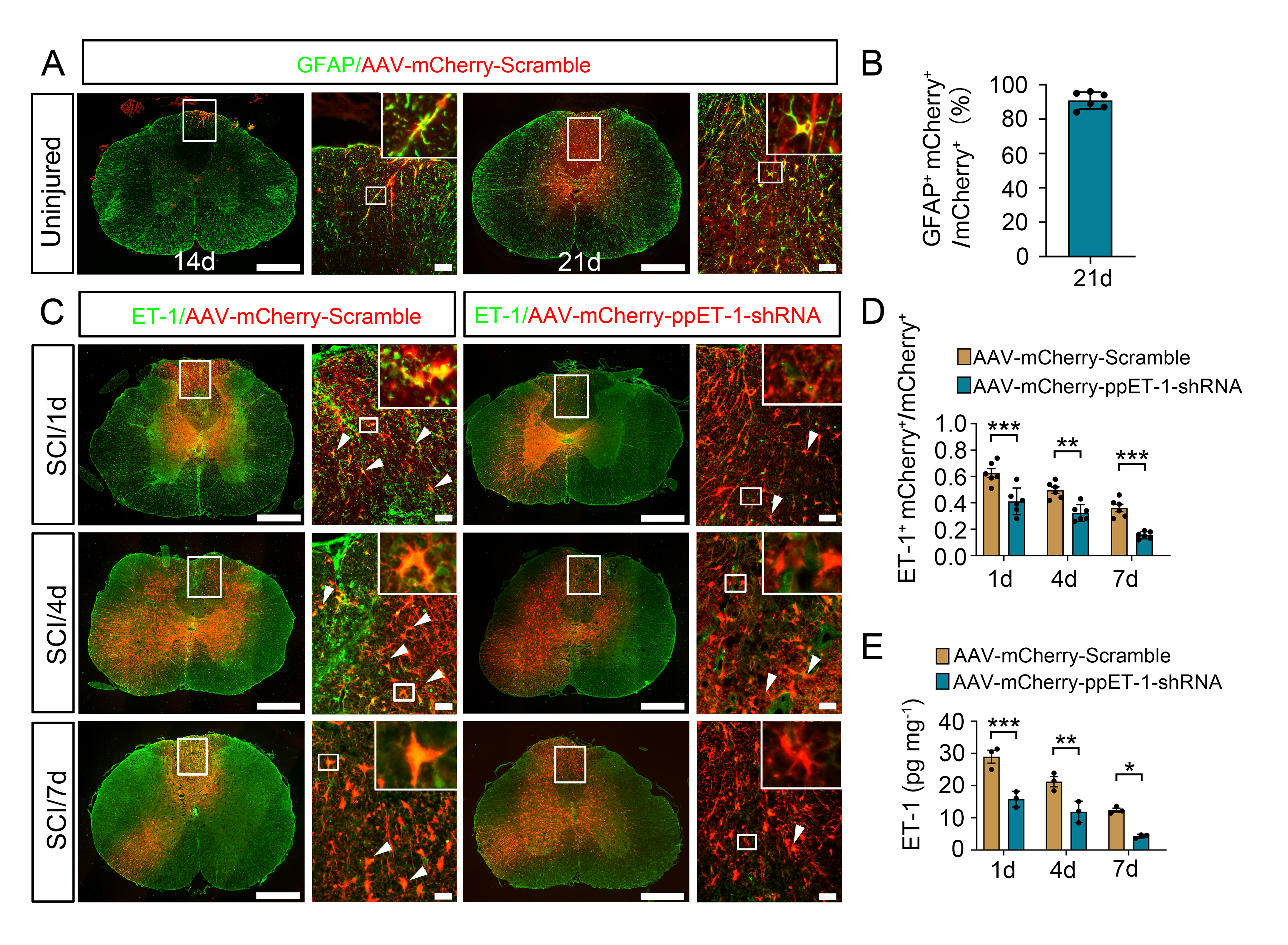


**Figure S2**. Effects of astrocyte-specific knockdown of ppET-1 expression on the ET-1 production following rat SCI. A, B) Immunostaining showed colocalization of mCherry and GFAP-positive astrocytes at the surgical site of the cord at 14 d and 21 d, following injection of 10 μL AAV5-GFAP-mCherry-Scramble (1×10^13^ vg mL^-1^). Scale bars, 500 μm and 50 μm in magnification. B) Quantification data as shown in (A). Data are expressed as mean ± SEM, *n* = 6. C) Immunostaining showed colocalization of ET-1 with mCherry-positive cells at the lesion site of the cord at 1 d, 4 d, and 7 d, following SCI, after pre-injection of 10 μL AAV5-GFAP-mCherry-ppET-1-shRNA (1×10^13^ vg mL^-1^) or AAV5-GFAP-mCherry-Scramble for 21 d. Scale bars, 500 μm and 50 μm in magnification. D) Quantification data as shown in (C). Data are expressed as mean ± SEM, n = 6. ***P* < 0.01, ****P* < 0.001, two-way analysis of variance followed by Sidak's *post hoc* test. E) ELISA measurement of ET-1 protein at lesion sites at 1 d, 4 d and 7 d following SCI, after pre-injection of 10 μL AAV5-GFAP-mCherry-ppET-1-shRNA (1×10^13^ vg mL^-1^) or AAV5-GFAP-mCherry-Scramble for 21 d. Data are expressed as mean ± SEM, *n* = 6, **P* < 0.05, ***P* < 0.01, ****P* < 0.001, two-way analysis of variance followed by Sidak's *post hoc* test.


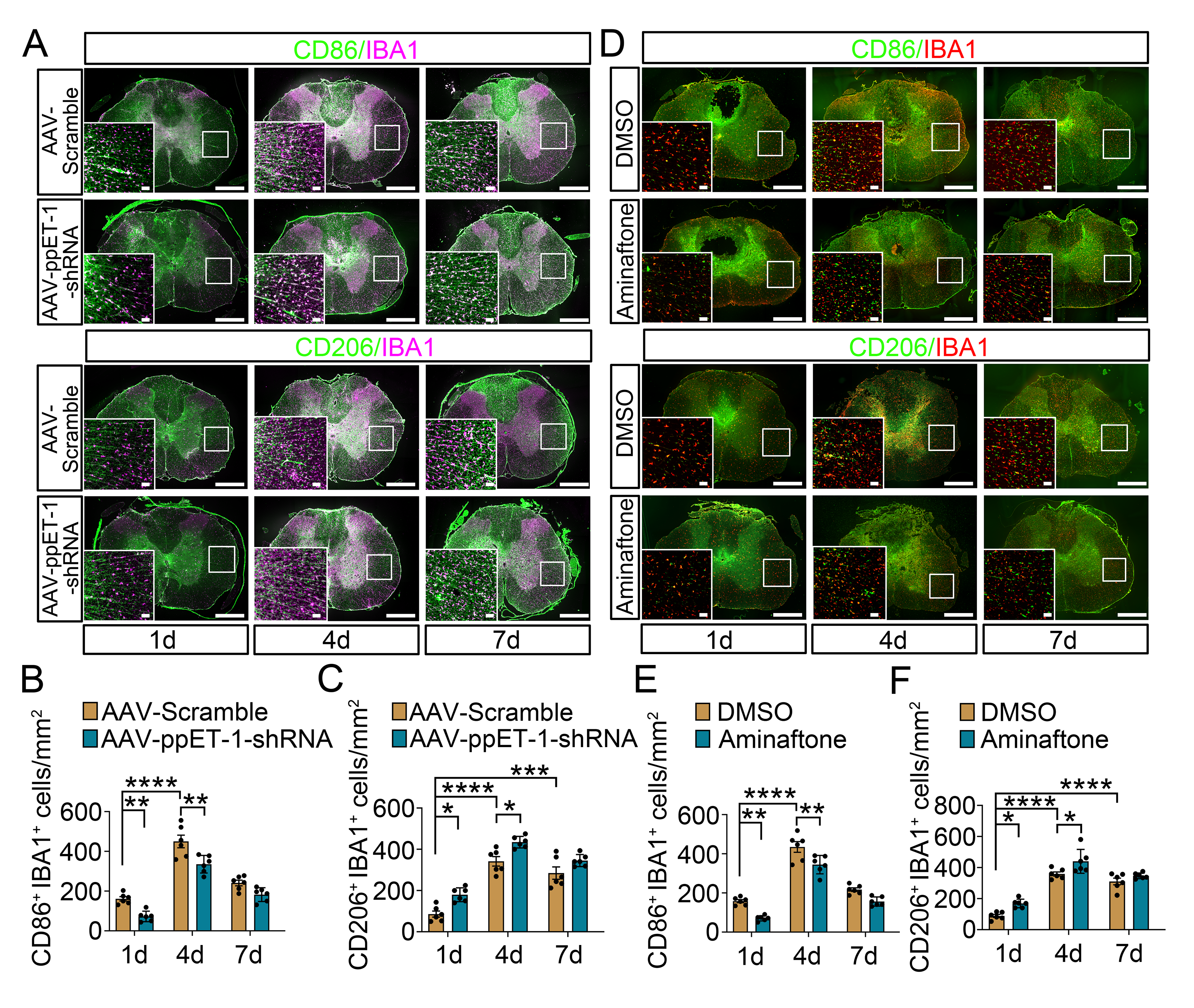


**Figure S3.** Analysis of effects of astrocytic ET-1 system inactivation on the polarization of microglia phenotypes following SCI. A) Immunostaining of CD86^+^ IBA1^+^ and CD206^+^ IBA1^+^ cells at the lesion site of the cord at 1 d, 4 d and 7 d after SCI, respectively, following pre-injection of 10 μL AAV5-GFAP-mCherry-ppET-1-shRNA (1×10^13^ vg mL^-1^) or AAV5-GFAP-mCherry-Scramble for 21 d. The rectangle indicates the region magnified. Scale bars, 500 μm and 50 μm in magnification. B, C) Quantification data as shown in (A). Data are expressed as mean ± SEM, *n* = 6, **P* < 0.05, ***P* < 0.01, ****P* < 0.001, *****P* < 0.0001, two-way analysis of variance followed by Sidak's *post hoc* test. D**)** Immunostaining of CD86^+^ IBA1^+^ and CD206^+^ IBA1^+^ cells at the lesion site of the cord at 1 d, 4 d and 7 d, respectively, following injection of 4.5 μL ET-1 inhibitor Aminaftone (500 μg kg^-1^). The DMSO (0.1%) was used as vehicle. The rectangle indicates the region magnified. Scale bars, 500 μm and 50 μm in magnification. E, F) Quantification data as shown in (D). Data are expressed as mean ± SEM, *n* = 6, **P* < 0.05, ***P* < 0.01, *****P* < 0.0001, two-way analysis of variance followed by Sidak's *post hoc* test.


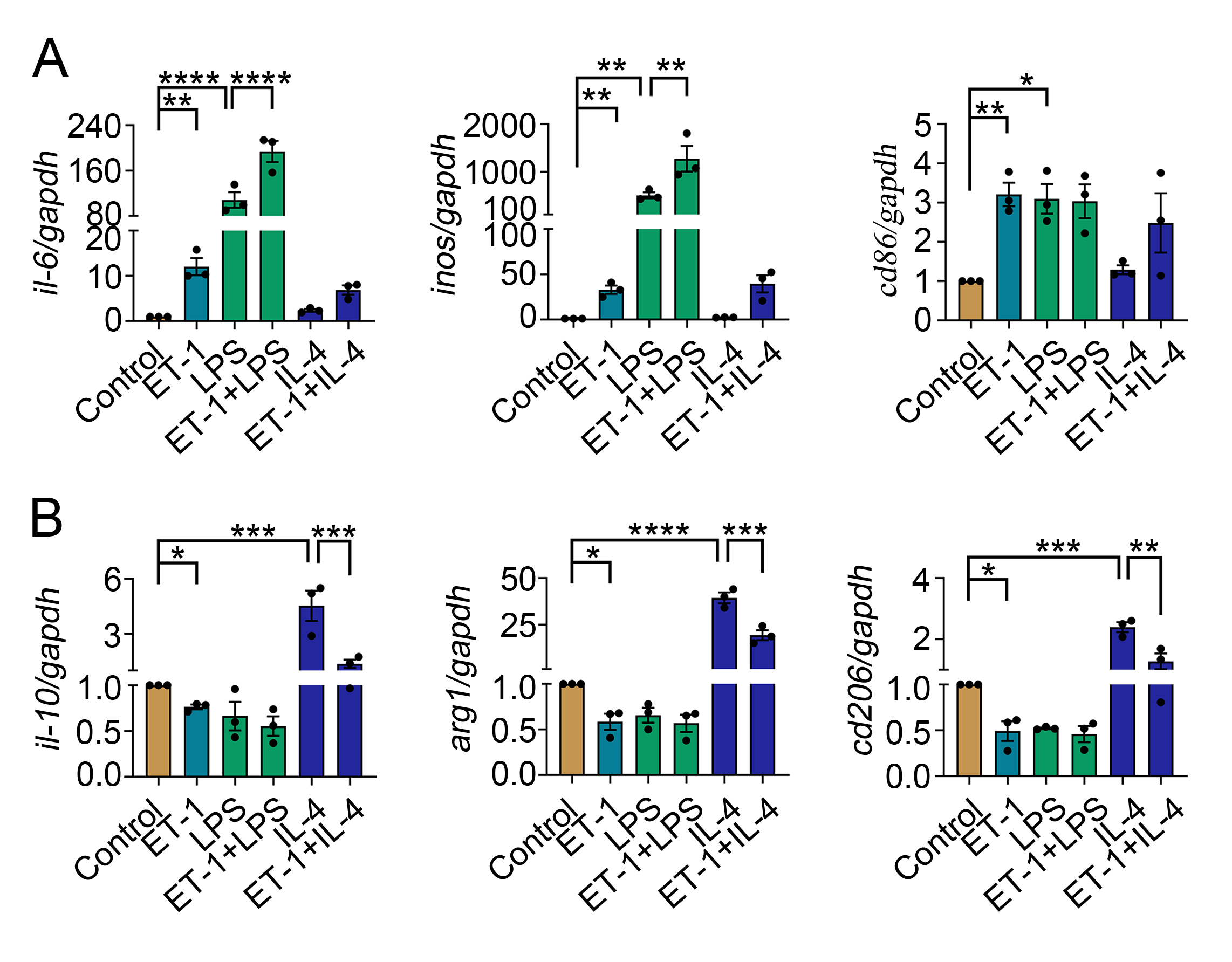


**Figure S4.** Analysis of ET-1 effects on LPS- or IL-4-induced microglia phenotype polarization. A**,** B) qRT-PCR assays for M1 and M2 phenotype-related molecules following microglia stimulated with 0.5 µg mL^-1^ LPS or 20 ng mL^-1^ IL-4 in the presence of 200 ng mL^-1^ ET-1 for 24 h. Data are expressed as mean ± SEM, *n* = 3, **P* < 0.05, ***P* < 0.01, ****P* < 0.001, *****P* < 0.0001, one­way analysis of variance followed by Sidak's *post hoc* test. Quantities were normalized to endogenous *gapdh*.


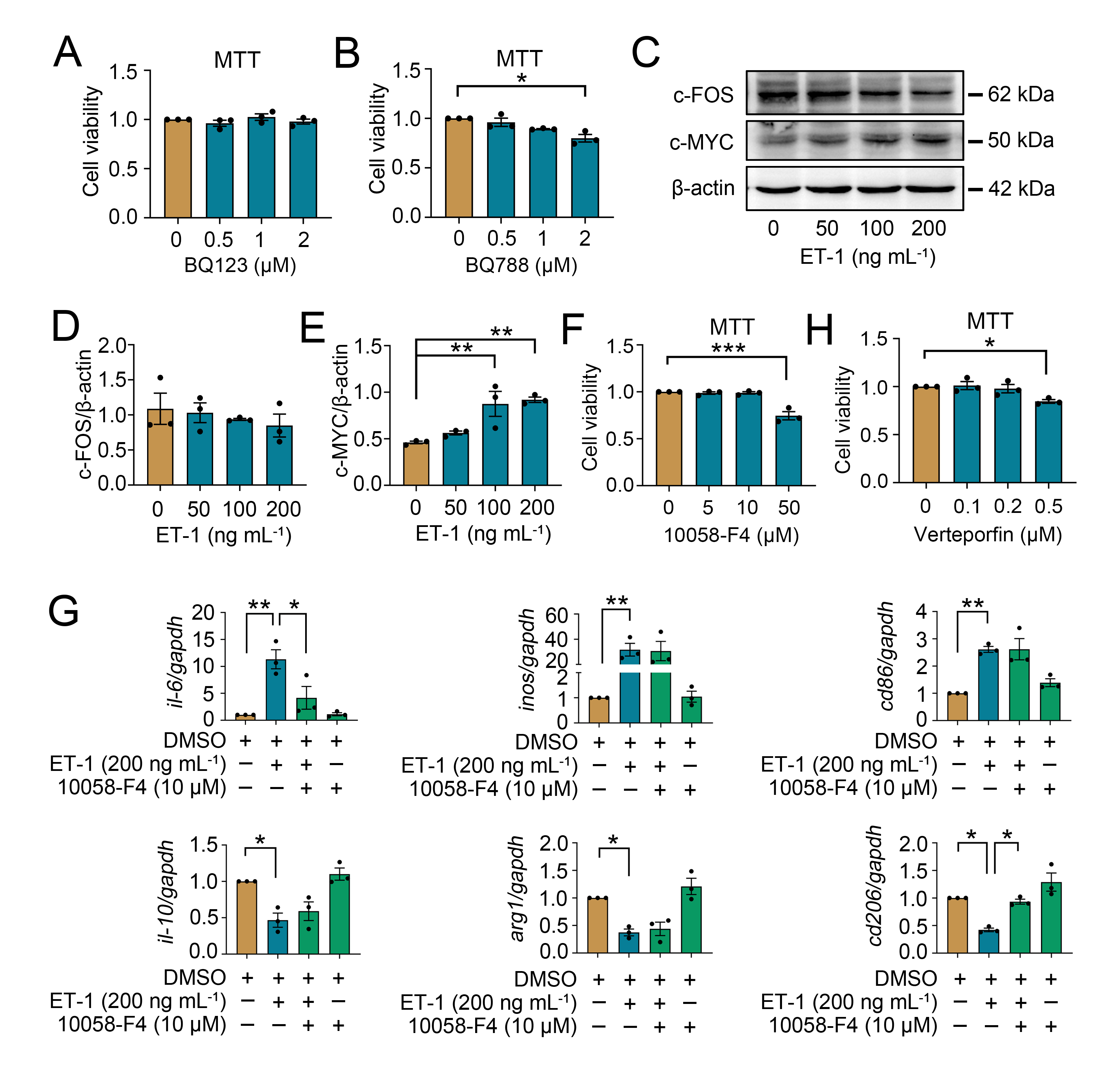


**Figure S5.** Examination of c-FOS or c-MYC roles on ET-1-mediated microglial polarization. A, B) MTT assays for cell viability after treatment of microglia with ET_A_ inhibitor BQ123 or ET_B_ inhibitor BQ788. Data are expressed as mean ± SEM, *n* = 3, **P* < 0.05, one­way analysis of variance followed by Dunnett's *post hoc* test. C) Western blot analysis of transcription factor c-FOS and c-MYC following exposure of microglia to 0 - 200 ng mL^-1^ ET-1 for 24 h. D, E) Quantification data as shown in (C). Data are expressed as mean ± SEM, n = 3, ***P* < 0.01, one­way analysis of variance followed by Dunnett's *post hoc* test. Quantities were normalized to endogenous β-actin. F) MTT assays for cell viability after the microglia treatment with c-MYC inhibitor 10058-F4. Data are expressed as mean ± SEM, *n* = 3, ****P* < 0.001, one­way analysis of variance followed by Dunnett's *post hoc* test. G) qRT-PCR analysis of microglia M1 phenotype-related genes IL-6, iNOS and CD86, and M2 phenotype-related genes IL-10, Arg1 and CD206 following treatment of the cells with 10 μM c-MYC inhibitor 10058-F4 for 24 h in the presence of 200 ng mL^-1^ ET-1. Data are expressed as mean ± SEM, *n* = 3, **P* < 0.05, ***P* < 0.01, one­way analysis of variance followed by Sidak's *post hoc* test. Quantities were normalized to endogenous *gapdh.* H) MTT assay was used to detect the cell viability of microglia after treatment with the YAP inhibitor verteporfin. Data are expressed as mean ± SEM, *n* = 3, **P* < 0.05, one­way analysis of variance followed by Dunnett's *post hoc* test.

**
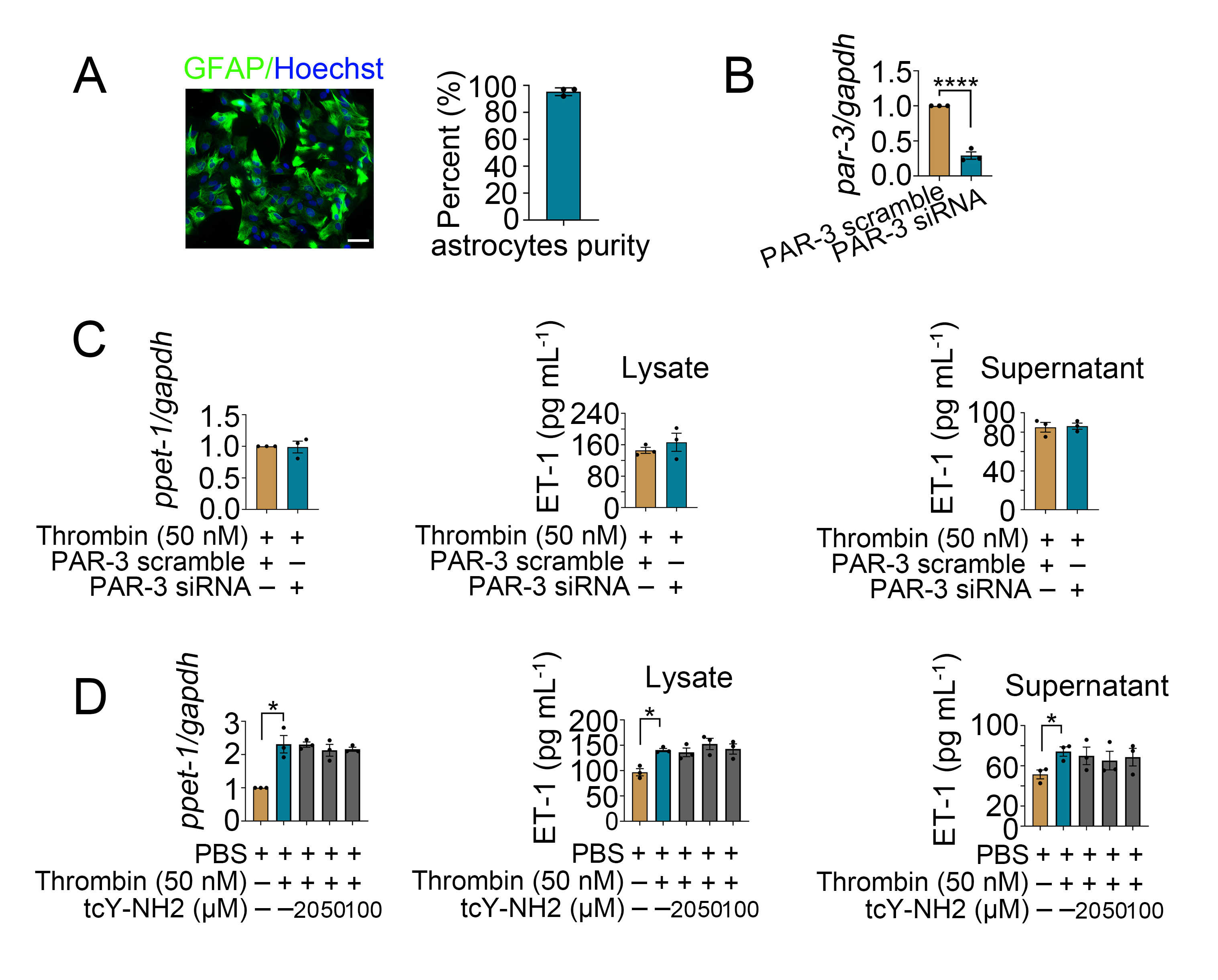
**

**Figure S6.** Effects of PAR-3 or PAR-4 interference on the thrombin-induced astrocytic production of ET-1. A) Isolation and purification of primary astrocytes from rat spinal cord, with purity over 95%. B) The interference efficiency of siRNA oligonucleotide for PAR-3 was measured by qRT-PCR. Scrambles were used as control. Data are expressed as mean ± SEM, *n* = 3, *****P* < 0.0001, two-tailed unpaired Student's *t*-test. Quantities were normalized to endogenous *gapdh*. C) The expression of ppET-1 and ET-1 in the astrocytes was measured by qRT-PCR and ELISA after PAR-3 knockdown for 48 h, followed by stimulation with 50 nM thrombin for 12 h. Scrambles were used as control. Data are expressed as mean ± SEM, *n* = 3, two-tailed unpaired Student's *t*-test. D) The expression of ppET-1 and ET-1 was determined by qRT-PCR and ELISA following treatment of the astrocytes with 20 - 100 μM PAR-4 inhibitor tcY-NH2 for 12 h in the presence of 50 nM thrombin. Data are expressed as mean ± SEM, *n* = 3, **P* < 0.05, one­way analysis of variance followed by Sidak's *post hoc* test.


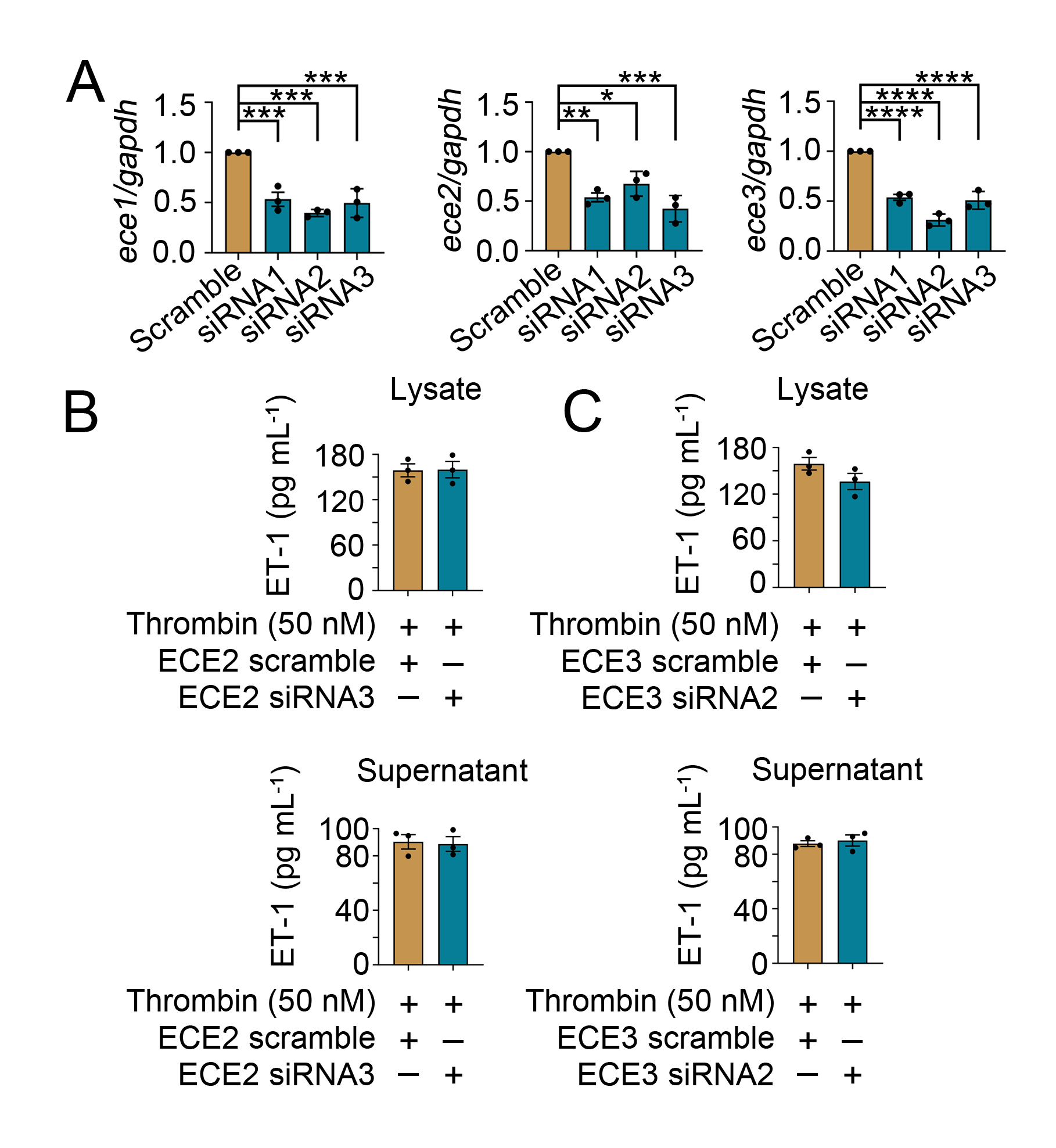


**Figure S7.** Determination of ECE2 and ECE3 effect on the processing of thrombin-induced astrocytic mature ET-1. A) qRT-PCR analysis of the interference efficiency of ECE1, ECE2 and ECE3 siRNAs, respectively. Scrambles were used as control. Data are expressed as mean ± SEM, *n* = 3, **P* < 0.05, ***P* < 0.01, ****P* < 0.001, *****P* < 0.0001, one­way analysis of variance followed by Dunnett's *post hoc* test. Quantities were normalized to endogenous *gapdh*. B, C) ELISA measurement of the astrocytic ET-1 production after knockdown of ECE2 or ECE3 expression for 48 h, followed by treatment with 50 nM thrombin for 12 h. Data are expressed as mean ± SEM, *n* = 3, two-tailed unpaired Student's *t*-test.

**
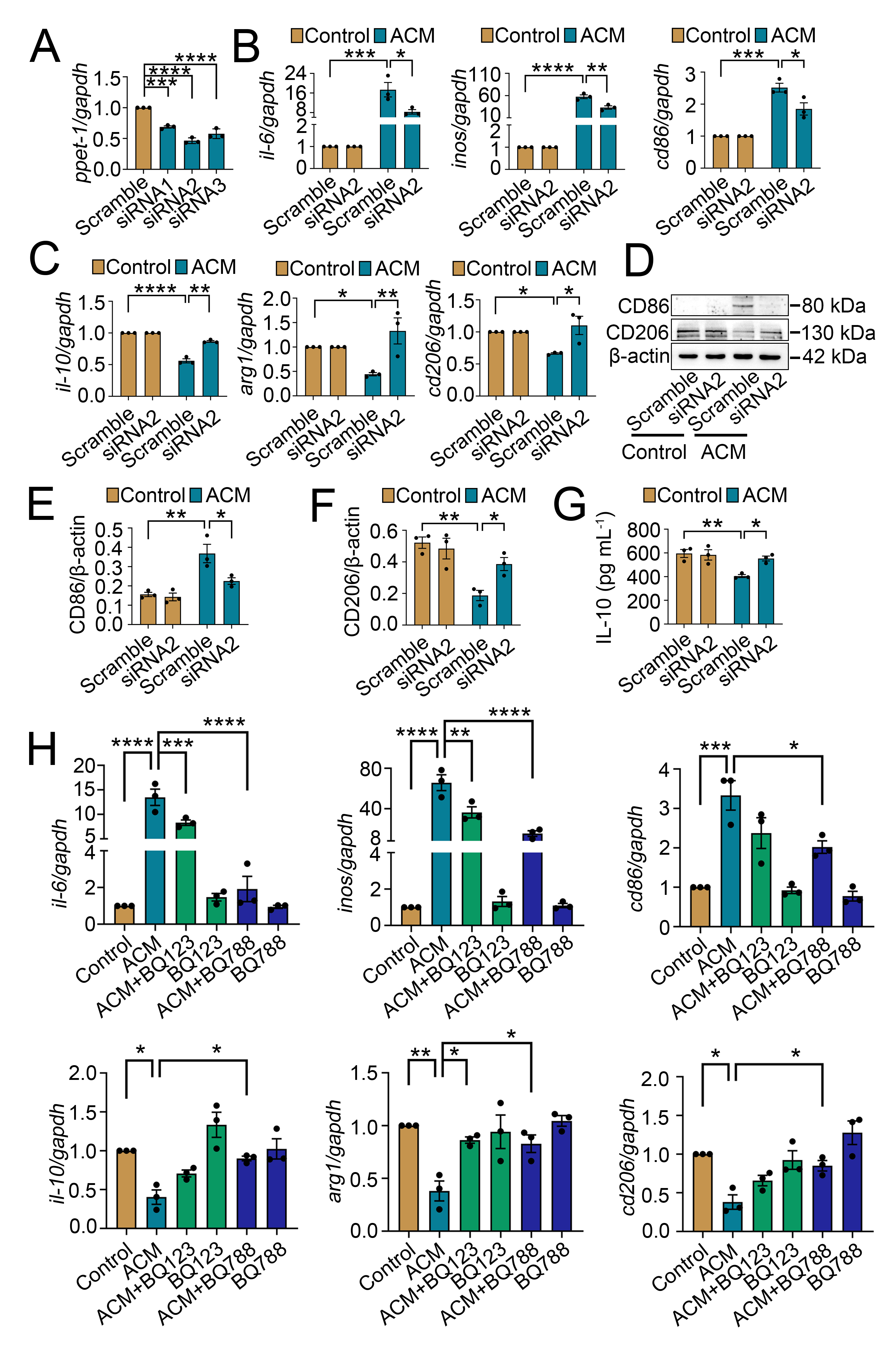
**

**Figure S8.** Effects of interfering thrombin-induced astrocytic production of ET-1 on the microglial M1 polarization. A) Determination of the interference efficiency of ppET-1 siRNAs by qRT-PCR. Scrambles were used as control. Data are expressed as mean ± SEM, *n* = 3, ****P* < 0.001, *****P* < 0.0001, one­way analysis of variance followed by Dunnett's *post hoc* test. Quantities were normalized to endogenous *gapdh*. B, C) Determination of M1 phenotype-related genes IL-6, iNOS and CD86, and M2 phenotype-related genes IL-10, Arg1 and CD206 after culture of microglia with the ACM for 24 h. The ACM was prepared from astrocytes with or without ppET-1 siRNA interference for 48 h, followed by treatment with 50 nM thrombin for 12 h. Quantities were normalized to endogenous *gapdh*. Data are expressed as mean ± SEM, *n* = 3, **P* < 0.05, ***P* < 0.01, ****P* < 0.001, *****P* < 0.0001, two-way analysis of variance followed by Tukey's *post hoc* test. D) Western blot analysis of CD86 and CD206 protein levels after treatment of microglia with the ACM for 24 h, the ACM was prepared from astrocytes with or without ppET-1 siRNA interference for 48 h, followed by treatment with 50 nM thrombin for 12 h. E, F) Quantification data as shown in (D). Quantities were normalized to endogenous β-actin. Data are expressed as mean ± SEM, *n* = 3, **P* < 0.05, ***P* < 0.01, two-way analysis of variance followed by Tukey's *post hoc* test. G) ELISA measurement of IL-10 after microglia treatment with the ACM for 24 h, the ACM was prepared from astrocytes with or without ppET-1 siRNA interference for 48 h, followed by treatment with 50 nM thrombin for 12 h. Data are expressed as mean ± SEM, *n* = 3, **P* < 0.05, ***P* < 0.01, two-way analysis of variance followed by Tukey's *post hoc* test. H) Determination of IL-6, iNOS, CD86, IL-10, Arg1 and CD206 following treatment of microglia with 1 μM ET_A_ inhibitor BQ123 or 1 μM ET_B_ inhibitor BQ788 for 24 h in the presence of the ACM. The ACM was prepared by treatment of astrocytes with 50 nM thrombin for 12 h. Data are expressed as mean ± SEM, *n* = 3, **P* < 0.05, ***P* < 0.01, ****P* < 0.001, *****P* < 0.0001, one­way analysis of variance followed by Sidak's *post hoc* test. Quantities were normalized to endogenous *gapdh*.


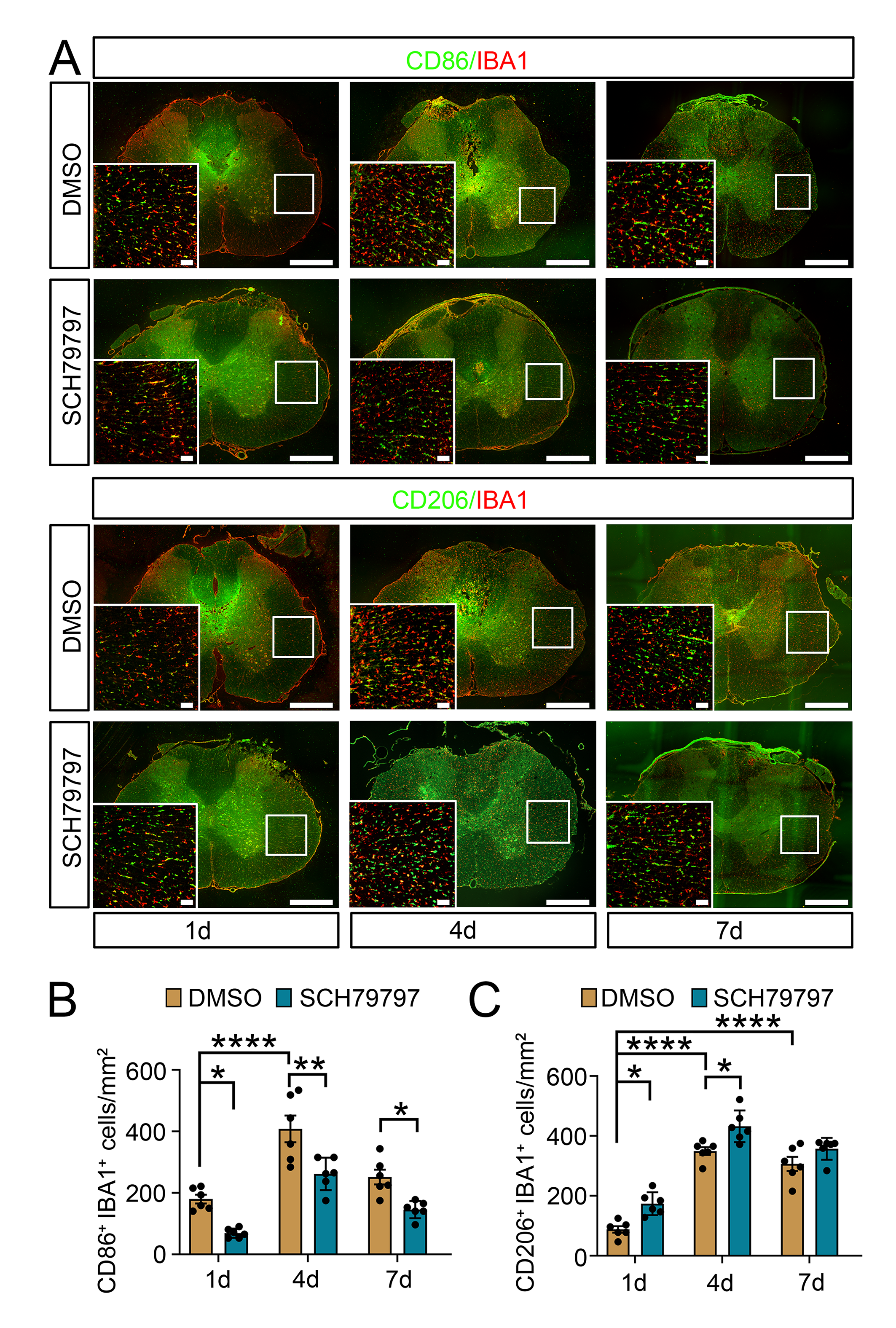


**Figure S9.** Analysis of effects of PAR-1 inactivation on the polarization of microglia phenotypes following SCI. A) Immunostaining of CD86^+^ IBA1^+^ and CD206^+^ IBA1^+^ microglia at the lesion site of the cord at 1 d, 4 d and 7 d following intrathecal injection of 4.5 μL PAR-1 inhibitor SCH79797 (50 μg kg^-1^). The DMSO (0.1%) was used as vehicle. Rectangles indicate the region magnified. Scale bars, 500 μm and 50 μm in magnification. B, C) Quantification data as shown in (A). Data are expressed as mean ± SEM, *n* = 6, **P* < 0.05, ***P* < 0.01, *****P* < 0.0001, two-way analysis of variance followed by Sidak's *post hoc* test.

Table S1: Specific primer sequences.

| Gene | Forward primer | Reverse primer |
| --- | --- | --- |
| *ppet1* | 5'-GCCATCAGCAACAGCATCAAG-3' | 5'-GTCCTCTGCCAGTCTGAACAA-3' |
| *ppet2* | 5'-ACTCTGCCTGTGCCACCTTCT-3' | 5'-GCCGTAGCCTGACAAATTGGAG-3' |
| *ppet3* | 5'-ACTACTGCCACCTGGACATCAT-3' | 5'-CGTAAGCGTGTCTGTGGAGAAG-3' |
| *ece1* | 5'-TTCGCCCAACGCCTTGAACTTT-3' | 5'-ACTCGGTCTGCTGCTTGAATGC-3' |
| *ece2* | 5'-AGCGGCGTGATGAGGAGAAGAT-3' | 5'-CACCACCACAGGCTCAGAATCG-3' |
| *ece3* | 5'-CAACAACAGCAGCAGGCACAAG-3' | 5'-AGGGTCTGGGAGGGACTCAAAG-3' |
| *eta* | 5'-CGTGAAGGACTGGTGGCTCTT-3' | 5'-CTGCTTGAGGTGTTCGCTGAG-3' |
| *etb* | 5'-TCTACTTCTGCTTGCCGCTAG-3' | 5'-GCCACTTCTCGTCTCTGCTT-3' |
| *il-6* | 5'-CTTCCAGCCAGTTGCCTTCTTG-3' | 5'-TGGTCTGTTGTGGGTGGTATCC-3' |
| *inos* | 5'-CTGAGACAAGGAAGTCGGAAG-3' | 5'-GTCACCACCAGCAGTAGTTG3' |
| *cd86* | 5'-CAGATGCTGTTCCTGTGAAGAG-3' | 5'-AAGTCGTAGAGCCTGGTTATCC-3' |
| *il-10* | 5'-GCTGGACAACATACTGCTGAC-3' | 5'-TGCTCCACTGCCTTGCTT-3' |
| *arg1* | 5'-TGGAGACCACAGTATGGCAAT-3' | 5'-CACGATGTCCTTGGCAGATATG-3' |
| *cd206* | 5'-GACAGACGGACGAGGAGTTC-3' | 5'-CCACCAATCACAACAACACAGT-3' |
| *gapdh* | 5'-ACAGCAACAGGGTGGTGGAC-3' | 5'-TTTGAGGGTGCAGCGAACTT-3' |
