# Supplementary material for: Astrocytic ET‐1 System Determines Microglia Phenotype Following Spinal Cord Injury
Source: Adv Sci (Weinh). 2025 May 30;12(31):e07215. doi: 10.1002/advs.202507215 (PMC12376558; doi:10.1002/advs.202507215)

ADVANCED SCIENCE
Open Access

Supporting Information

for *Adv. Sci.*, DOI 10.1002/advs.202507215

Astrocytic ET-1 System Determines Microglia Phenotype Following Spinal Cord Injury

*Bingqiang He, Si Xu, Mengdi Li, Hui Li, Shaolan Li, Li Niu, Honghua Song, Rixin Cai, Yue Zhou, Zhilong Cao, Yingjie Wang and Yongjun Wang**

Figure 3B. ETs

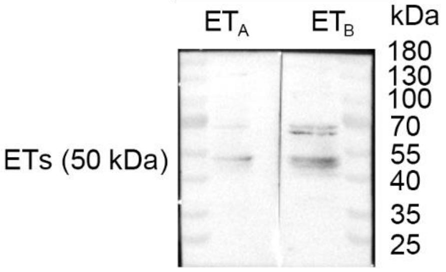

Figure 3H. ETs

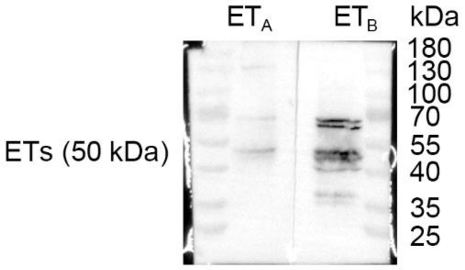

Figure 3B. β-actin

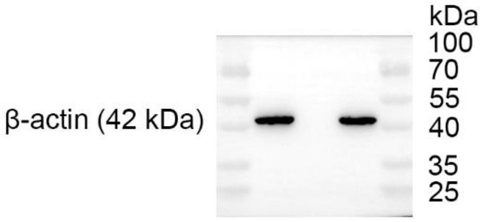

Figure 3H. β-actin

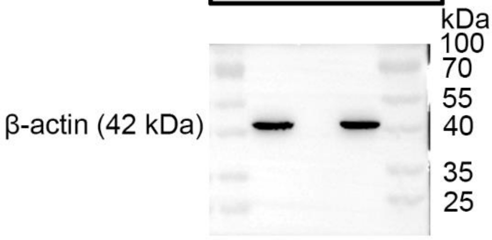

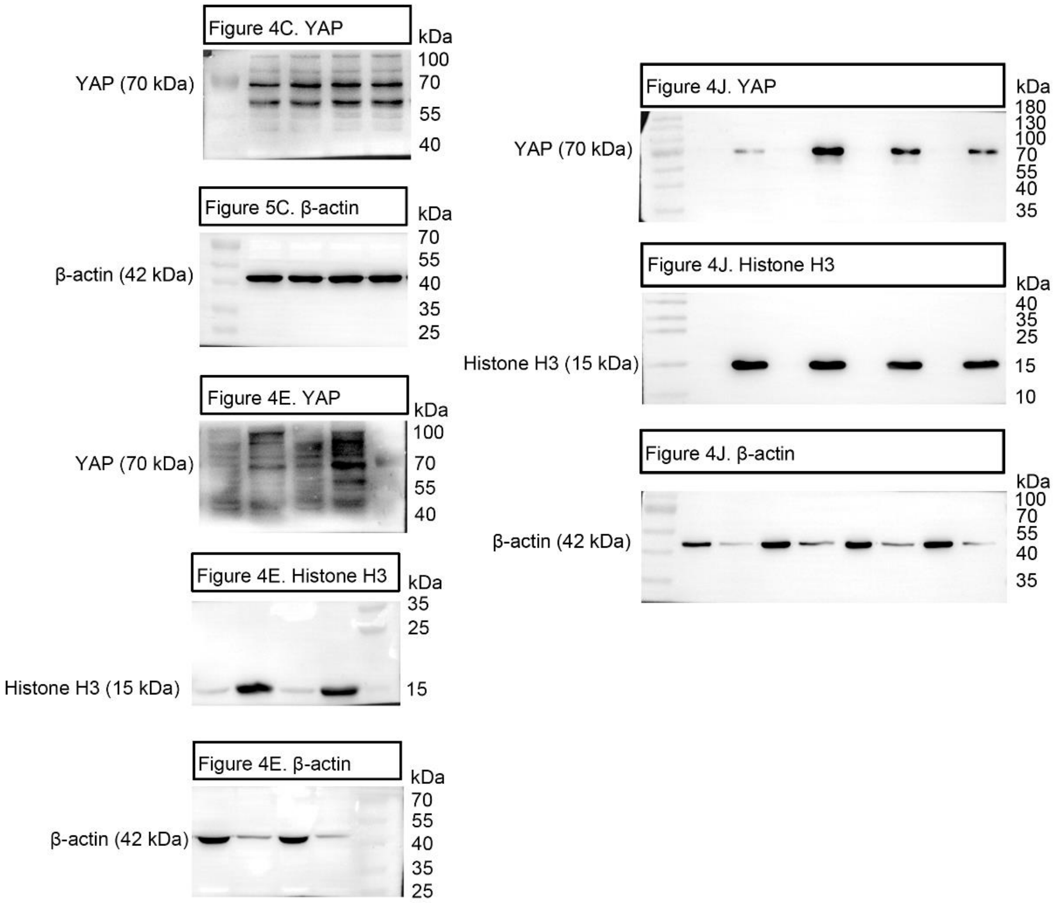
Figure 4C. YAP
kDa
100
70
55
40
YAP (70 kDa)
Figure 5C. β-actin
kDa
70
55
40
35
25
β-actin (42 kDa)
Figure 4E. YAP
kDa
100
70
55
40
YAP (70 kDa)
Figure 4E. Histone H3
kDa
35
25
15
Histone H3 (15 kDa)
Figure 4E. β-actin
kDa
70
55
40
35
25
β-actin (42 kDa)
Figure 4J. YAP
kDa
180
130
100
70
55
40
35
YAP (70 kDa)
Figure 4J. Histone H3
kDa
40
35
25
15
10
Histone H3 (15 kDa)
Figure 4J. β-actin
kDa
100
70
55
40
35
β-actin (42 kDa)

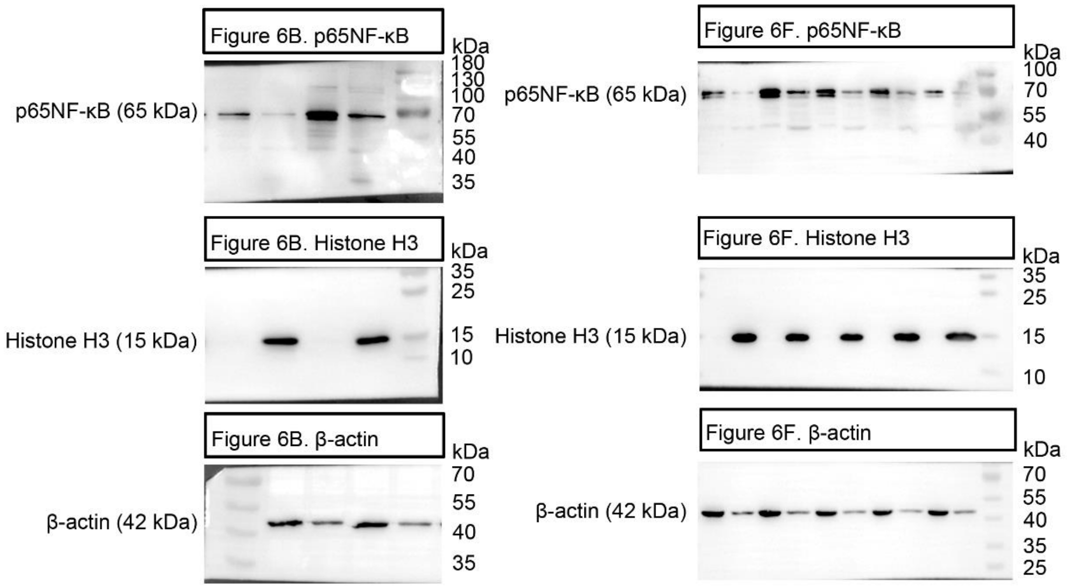
Figure 6B. p65NF-κB
p65NF-κB (65 kDa)
kDa
180
130
100
70
55
40
35
Figure 6B. Histone H3
Histone H3 (15 kDa)
kDa
35
25
15
10
Figure 6B. β-actin
β-actin (42 kDa)
kDa
70
55
40
35
Figure 6F. p65NF-κB
p65NF-κB (65 kDa)
kDa
100
70
55
40
Figure 6F. Histone H3
Histone H3 (15 kDa)
kDa
35
25
15
10
Figure 6F. β-actin
β-actin (42 kDa)
kDa
70
55
40
35
25

Figure 7B. YAP

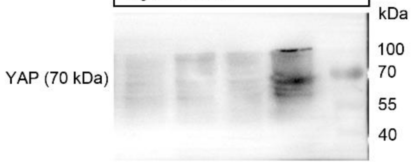

Figure 7B. Histone H3

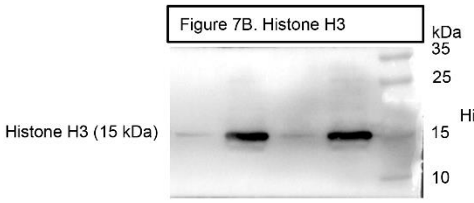

Figure 7B. β-actin

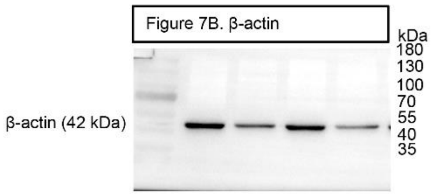

Figure 7F. YAP

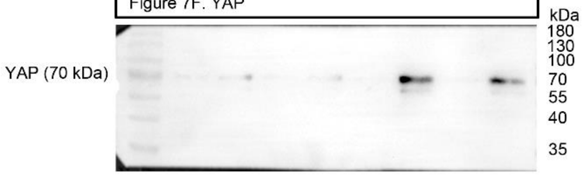

Figure 7F. Histone H3

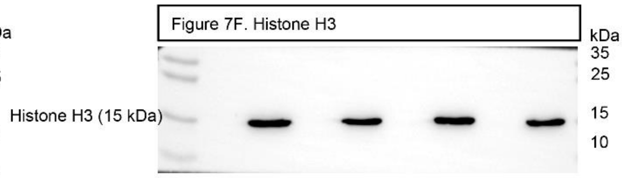

Figure 7F. β-actin

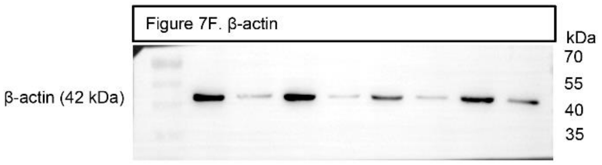

Figure 7I. YAP

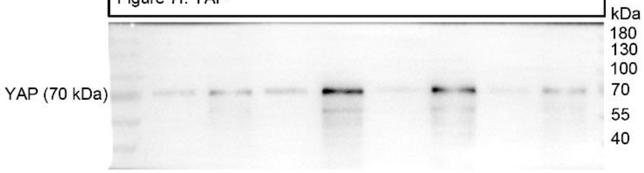

Figure 7I. Histone H3

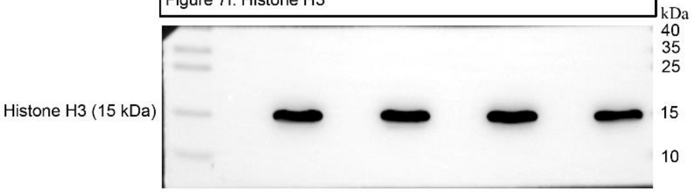

Figure 7I. β-actin

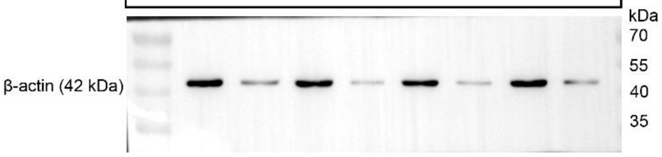

Figure S5C. c-FOS

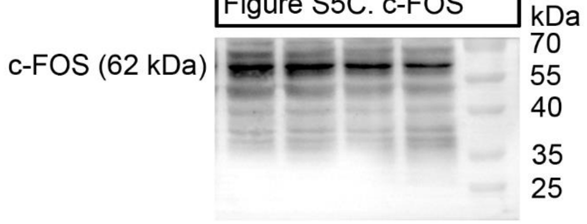

Figure S5C. c-MYC

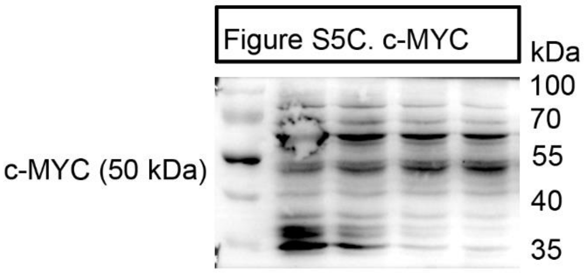

Figure S5C. β-actin

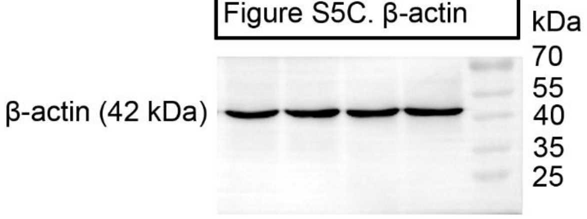

Figure S8D. CD86

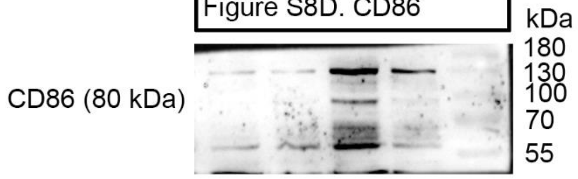

Figure S8D. CD206

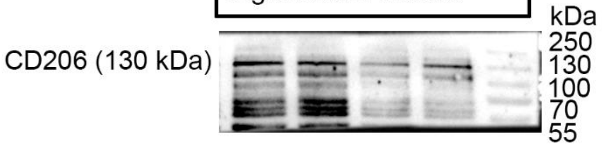

Figure S8D. β-actin

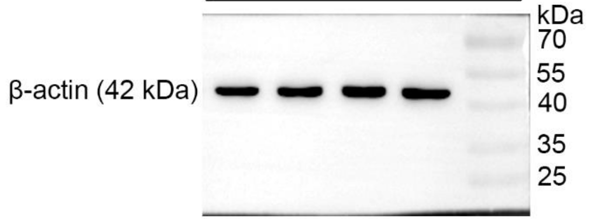

Supplement: Supplementary file 2 — Supporting Information [file ADVS-12-e07215-s001.pdf]
